# Inhibition of CARM1‐Mediated Methylation of ACSL4 Promotes Ferroptosis in Colorectal Cancer

**DOI:** 10.1002/advs.202303484

**Published:** 2023-11-09

**Authors:** Shengjie Feng, Zejun Rao, Jiakun Zhang, Xiaowei She, Yaqi Chen, Kairui Wan, Haijie Li, Chongchong Zhao, Yongdong Feng, Guihua Wang, Junbo Hu, Xuelai Luo

**Affiliations:** ^1^ GI Cancer Research Institute Tongji Hospital Huazhong University of Science and Technology Wuhan 430030 P. R. China; ^2^ The HIT Center for Life Sciences Harbin Institute of Technology Harbin 150001 China

**Keywords:** ACSL4 methylation, CARM1, ferroptosis

## Abstract

Ferroptosis, which is caused by iron‐dependent accumulation of lipid peroxides, is an emerging form of regulated cell death and is considered a potential target for cancer therapy. However, the regulatory mechanisms underlying ferroptosis remain unclear. This study defines a distinctive role of ferroptosis. Inhibition of CARM1 can increase the sensitivity of tumor cells to ferroptosis inducers in vitro and in vivo. Mechanistically, it is found that ACSL4 is methylated by CARM1 at arginine 339 (R339). Furthermore, ACSL4 R339 methylation promotes RNF25 binding to ACSL4, which contributes to the ubiquitylation of ACSL4. The blockade of CARM1 facilitates ferroptosis and effectively enhances ferroptosis‐associated cancer immunotherapy. Overall, this study demonstrates that CARM1 is a critical contributor to ferroptosis resistance and highlights CARM1 as a candidate therapeutic target for improving the effects of ferroptosis‐based antitumor therapy.

## Graphical abstract

This discovery suggests that CARM1‐mediated ACSL4 R339 methylation is a vital regulatory mechanism for ferroptosis resistance and tumor progression. Additionally, CARM1 may be highlighted as a candidate therapeutic target for improving the effects of ferroptosis‐based antitumor therapy.

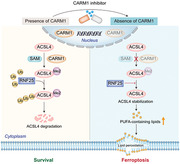

## 1 Introduction

Ferroptosis is a genetically encoded form of programmed cell death characterized by abnormal cellular metabolism and accumulation of iron‐dependent lipid peroxidation.^[^ ^1^, ^2^, ^3^ ^]^ It differs from the classical cell death processes, including apoptosis, necrosis, and pyroptosis. Accumulating evidence indicates that ferroptosis is associated with various pathological conditions and human diseases, such as tissue damage, inflammation, neurodegeneration, and cancer.^[^ ^4^, ^5^, ^6^, ^7^, ^8^ ^]^ Notably, ferroptosis is involved in tumor suppression through multiple mechanisms. Calcium‐independent phospholipase A2β inhibits ferroptosis by hydrolyzing peroxidized phospholipids.^[^ ^9^ ^]^ Additionally, TP53 downregulates the expression of the system Xc^−^ subunit SLC7A11, which promotes ferroptosis and contributes to its tumor‐suppressive function.^[^ ^10^ ^]^ Moreover, the NF2‐YAP, p62‐Keap1‐NRF2, and polyunsaturated fatty acid (PUFA) metabolism signaling pathways are involved in the regulation of ferroptosis.^[^ ^11^, ^12^, ^13^ ^]^ Recent reports have shown that ferroptosis may enhance the antitumor efficacy of immunotherapies in cancer.^[^ ^14^, ^15^ ^]^ Therefore, an in‐depth investigation of the mechanism of tumor ferroptosis and its regulation is crucial and may be of considerable aid in guiding clinical targeted therapy.

Accumulation of lipid peroxidation products is a significant feature of ferroptosis and is regulated by the generation and elimination of lipid peroxides.^[^ ^16^ ^]^ Long‐chain fatty acid‐CoA ligases (ACSLs) are vital enzymes that have been widely studied in fatty acid metabolism. Studies on the mechanism of ferroptosis have provided evidence that ACSL4 critically contributes to ferroptosis sensitivity.^[^ ^17^, ^18^ ^]^ Functionally, ACSL4 catalyzes the conversion of PUFAs, especially arachidonic acid (AA) (C20:4) and adrenic acid (AdA) (C22:4), to their active form acyl‐CoA.^[^ ^19^, ^20^ ^]^ After processing by lysophosphatidylcholine acyltransferase 3,^[^ ^13^ ^]^ the products are inserted into phospholipids of the plasma membrane, after which they sensitize cells to ferroptosis. Although the function of ACSL4 in facilitating ferroptosis has been confirmed, its upstream regulators are not well‐defined.

Post‐translational modifications (PTMs) of non‐histone proteins, such as phosphorylation, methylation, acetylation, and ubiquitylation, have been reported to affect protein stability, function, and interactions. As a prevalent PTM, protein arginine methylation is catalyzed by arginine methyltransferases (PRMTs). CARM1 (also known as PRMT4) catalyzes the formation of asymmetric dimethylarginine (me2a) and facilitates tumor progression through a variety of methods. CARM1 serves as a coactivator of transcription for estrogen alpha and androgen receptors in breast and prostate cancers.^[^ ^21^, ^22^ ^]^ It can also directly methylate PKM2 and activate aerobic glycolysis, thereby promoting breast cancer development.^[^ ^23^ ^]^ Moreover, targeting CARM1 can sensitize resistant tumors to immunotherapy and enhance antitumor T‐cell function.^[^ ^24^ ^]^ However, the role of CARM1 in ferroptosis remains unclear. In this study, we demonstrated that CARM1 directly interacts with ACSL4 and inhibits ferroptosis by increasing ACSL4 methylation levels. Moreover, we showed that R339 methylation of ACSL4 is crucial for its protein stability and interaction with RNF25, which explains the mechanism underlying CARM1‐induced ferroptosis‐suppressive effects. Thus, we concluded that targeting CARM1 could be a potential therapeutic strategy for tumor ferroptosis.

## 2 Results

### 2.1 CARM1 was Negatively Associated with Ferroptosis in Colorectal Cancer

**Figure 1:**
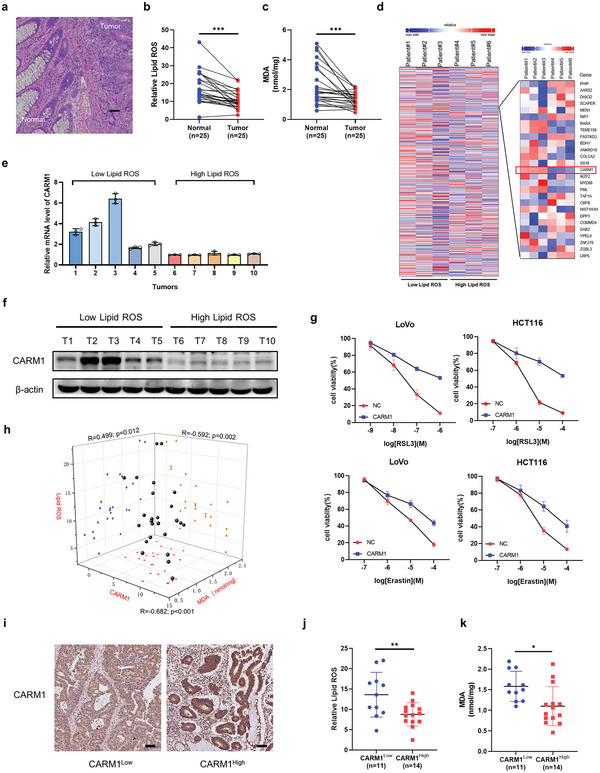
CARM1 was negatively associated with ferroptosis. a) HE staining of clinical specimens of colon cancer. Scale bar, 20 µm. b) Twenty‐five pairs of colorectal cancer (CRC) tissues and adjacent tissues were digested into single‐cell suspensions, and lipid ROS production was assayed via flow cytometry by using C11‐BODIPY after RSL3 treatment for 4 h (*n* = 25). c) Malondialdehyde (MDA) levels were detected by using a lipid peroxidation MDA assay kit in single‐cell suspensions treated with RSL3 for 4 h from 25 pairs of CRC tissues and adjacent tissues (*n* = 25). d) Heatmap of RNA‐seq using six patient tumors with different lipid ROS levels showing changes in gene expression, including CARM1. e) Quantitative real‐time PCR (qPCR) analysis of CARM1 mRNA levels in tumors from 10 patients. f) Western blot analysis of CARM1 in the same tissues as (e). g) Cell viability was assayed in vector‐ and CARM1‐overexpressing LoVo and HCT116 cells treated with the indicated doses of RSL3 and erastin for 24 h. h) Scatter plot of the immunohistochemistry (IHC) staining score for CARM1, lipid ROS, and MDA levels in CRC tissues (*n* = 25). All *p* values and *R* values were calculated with Spearman's *r* test. i) Representative results of immunohistochemical staining for CARM1 from 25 clinical CRC patients. Scale bars, 20 µm. j,k) Lipid ROS (left) and MDA (right) levels were compared in CARM1 high (CARM1 IHC score ≥ 6) and CARM1 low (CARM1 IHC score<6) groups. The data shown represent the mean ± SD. In (b) and (c), comparisons were made by using paired Student's *t*‐test, and in (j) and (k), comparisons were made by using the two‐tailed, unpaired Student's *t*‐test; ^*^ *p* < 0.05, ^**^ *p* < 0.01, ^***^ *p* < 0.001.

To assess the potential correlation between ferroptosis and the progression of colorectal cancer (CRC), we collected 25 pairs of CRC and adjacent tissues (**Figure** **1**a) and measured lipid peroxidation (lipid ROS) and malondialdehyde (MDA) levels to evaluate the ferroptosis level in these tissues treated with the ferroptosis inducer RSL3. The results showed that ferroptosis was weaker in carcinoma tissues than in para‐carcinoma tissues (Figure 1b,c). To screen the key factors regulating ferroptosis, we performed RNA‐seq using tumors from six patients with high lipid ROS levels and low lipid ROS levels; 461 genes were upregulated in the low lipid ROS group, whereas 680 genes were downregulated (Figure S1a, Supporting Information). Among these genes, we focused on methyltransferases, which have recently been reported to be involved in tumor progression. Our RNA‐seq data showed that CARM1 was upregulated in the low lipid ROS group, whereas PRDM8 and PRDM13 were downregulated (Figure 1d, Figure S1b, Supporting Information). However, compared with CARM1, the expression of PRDM8 and PRDM13 was negligible in the intestine (Figure S1c, Supporting Information). Therefore, we first verified CARM1 expression in CRC tissues. Quantitative real‐time PCR (qPCR) and Western blotting were performed to detect CARM1 expression at the mRNA and protein levels in 10 tumors from the high and low lipid ROS groups, and the results showed that CARM1 expression subsequently decreased as lipid ROS levels increased (Figure 1e,f).

To further verify the relationship between CARM1 and ferroptosis, we performed a cell viability assay using the CCK‐8 kit and found that CARM1 overexpression rescued the effects of RSL3‐ and erastin‐triggered ferroptosis (Figure 1g). In addition, we defined the level of CARM1 expression according to the immunohistochemical score of CARM1 and found that the expression of CARM1 was significantly negatively correlated with lipid ROS and MDA levels in CRC tumor tissues of patients (Figure 1h–k).

Consistent with CARM1 being considered a contributor to cancer progression, immunohistochemistry (IHC) staining and Western blotting results also showed that CARM1 was upregulated in CRC tissues (Figure S1d,e, Supporting Information). Subsequently, publicly available CRC expression profiles in TCGA‐COAD and GSE20916 datasets were analyzed, and CARM1 expression was elevated in both primary tumors and adenocarcinomas (Figure S1f, Supporting Information). Subsequently, IHC staining of a tissue chip containing 78 clinical specimens of malignant colon tumors was used to evaluate the survival curve, and patients with high CARM1 levels demonstrated significantly shorter overall survival (Figure S1g, Supporting Information). Collectively, these data suggested that CARM1 is negatively associated with ferroptosis in colorectal cells.

### 2.2 CARM1 Moderates Ferroptosis In Vivo and In Vitro

**Figure 2:**
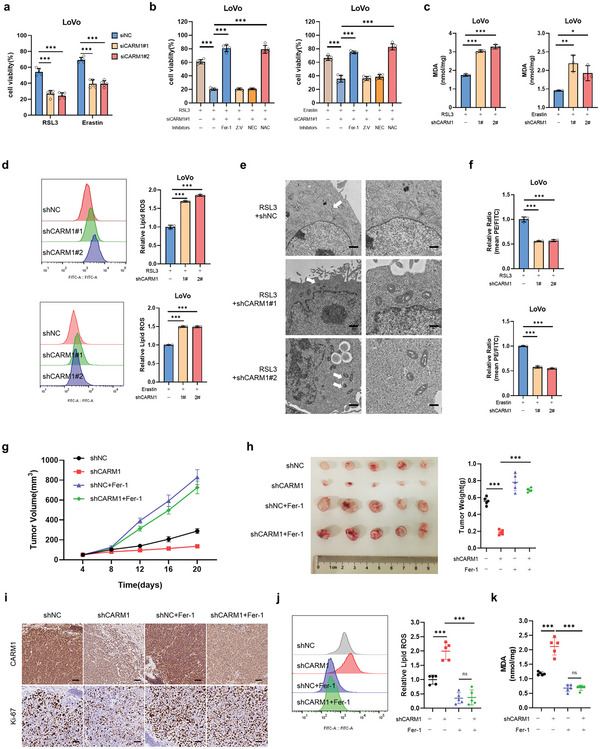
CARM1‐KD enhances ferroptotic cell death. a) Cell viability was measured in siNC and siCARM1 LoVo cells treated with 2.5 × 10^−6^ m RSL3 or 5 × 10^−6^ m erastin for 12 h (*n* = 5 independent experiments). b) Cell viability was measured in siNC and siCARM1 LoVo cells treated with cell death inhibitors and 2. 5 × 10^−6^ m RSL3 or 5 × 10^−6^ m erastin for 12 h. Fer‐1, 1 × 10^−6^ m ferrostatin‐1; NAC, 5 × 10^−3^ m; Nec, 2 × 10^−6^ m necrostatin‐1; Z‐V, 20 × 10^−6^ m Z‐VAD‐FMK (*n* = 5 independent experiments). c,d) Malondialdehyde (MDA) levels and relative lipid ROS were assayed in the indicated LoVo cells treated with 2. 5 × 10^−6^ m RSL3 or 5 × 10^−6^ m erastin for 12 h (*n* = 3 independent experiments). e) Transmission electron microscopy (TEM) images of the indicated LoVo cells subjected to RSL3 (2. 5 × 10^−6^ m) for 12 h. White arrows indicate mitochondria. Scale bars, left, 2 µm; right, 500 nm. f) The indicated stable LoVo cells were used to evaluate mitochondrial membrane potential via fluorescence staining of mitochondria with JC‐1 dye (*n* = 3 independent experiments). g) shNC and shCARM1 LoVo cells were subcutaneously injected into the mice. RSL3 was administered to all tumors with or without Fer‐1. Tumor volumes (*n* = 5) were calculated every 4 days, and the growth curve was drawn. h) Images of tumors from LoVo xenograft mice with altered treatments are shown, and the tumor weights (*n* = 5) of the subcutaneous xenografts were measured. i) Representative immunohistochemical images of CARM1 and Ki67 in tumor sections are shown. Scale bars, 20 µm. j,k) MDA levels and relative lipid ROS in tumor cells isolated from (h) were assayed (*n* = 5 independent experiments). The data shown represent the mean ± SD. Comparisons were made by using one‐way ANOVA with Tukey's test; ^*^ *p* < 0.05, ^**^ *p* < 0.01, ^***^ *p* < 0.001.

We examined whether CARM1 plays a crucial role in ferroptosis. As expected, CARM1 knockdown considerably aggravated cell death induced by ferroptosis inducers (such as RSL3 and erastin), and cell death could be fully rescued by ferrostatin‐1 (Fer‐1) or the antioxidant *N*‐acetyl‐cysteine (NAC), but not by an apoptosis inhibitor (Z‐V) or a necroptosis inhibitor (NEC) (**Figure** **2**a,b, Figure S2a–c, Supporting Information). Therefore, we constructed stable CARM1 shRNA‐CARM1 (shCARM1)‐expressing LoVo and HCT116 cell lines by using lentiviruses containing three altered shRNA sequences against CARM1 (Figure S2d, Supporting Information). These stable cell lines were used to explore the function of CARM1 in ferroptosis in subsequent studies. As reported in other studies, overexpression of CARM1 decreased ROS levels (Figure S2e,f, Supporting Information). In addition, CARM1 knockdown increased MDA and lipid peroxide levels, whereas CARM1 overexpression decreased MDA and lipid peroxide levels (Figure 2c,d, Figure S2g–j, Supporting Information). Because changes in the morphological features of mitochondria are one of the main characteristics of ferroptosis, transmission electron microscopy (TEM) was used to observe the condition of mitochondria in cells.^[^ ^25^ ^]^ The imaging results showed that the cells exhibited shrunken mitochondria with increased membrane density after CARM1 knockdown (Figure 2e). Moreover, fluorescence staining of mitochondria with JC‐1 dye showed reduced red fluorescence and increased green fluorescence in CARM1 knockdown cells, which was consistent with mitochondrial damage (Figure 2f, Figure S2k, Supporting Information). Notably, CARM1 overexpression eliminated this damage (Figure S2l, Supporting Information). To verify the role of CARM1 in vivo, a mouse xenograft model was constructed by using stable shNC and shCARM1 LoVo cell lines in nude mice, and tumors were treated with RSL3 with or without the ferroptosis inhibitor Fer‐1. The tumor growth curve demonstrated that tumors in the shCARM1 group grew more slowly, whereas Fer‐1 treatment significantly increased the growth rate of the tumor; the same results were observed with IHC staining for Ki‐67 (Figure 2g,i). After 20 days, the mice were sacrificed and the xenografts were collected. The xenografts formed by shCARM1 cells were smaller and lighter, and these effects were reversed by Fer‐1 (Figure 2h). Moreover, xenografts formed by shCARM1 cells demonstrated higher MDA and lipid peroxide levels than those formed by shNC cells; however, these effects were reversed by Fer‐1, as expected (Figure 2j,k). Overall, these results showed that CARM1 exerts ferroptosis‐suppressive effects in vivo and in vitro.

### 2.3 ACSL4 Mediates the Ferroptosis Function of CARM1

**Figure 3:**
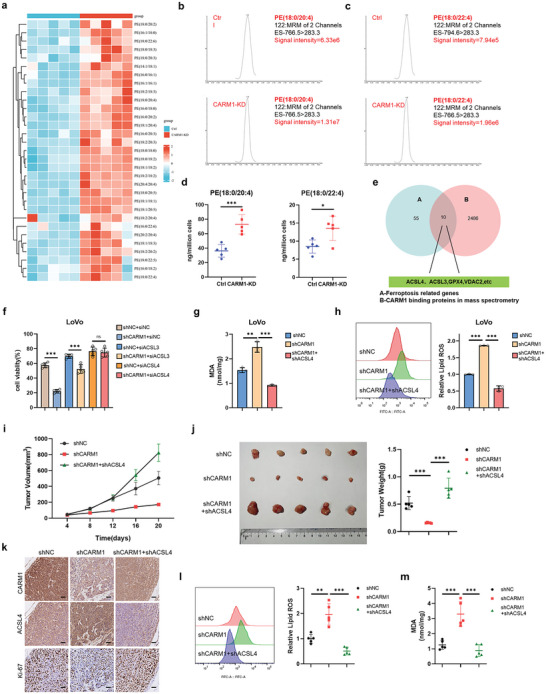
The ferroptosis function of CARM1 is mediated by ACSL4. a) Heatmap of all of the major phosphatidylethanolamine (PE) species in Ctrl and CARM1‐KD LoVo cells. Each PE species was normalized to the corresponding mean value. b,c) Representative EIC images of Ctrl and CARM1‐KD LoVo cells on two major molecular species of PE (PE [18:0/20:4] and PE [18:0/22:4]), representing substrates for oxygenation during ferroptosis. d) The contents of PE (18:0/20:4) and PE (18:0/22:4) in Ctrl and CARM1‐KD LoVo cells via LC‒MS (*n* = 5 independent experiments). e) Screening strategy for predicting the possible substrate of CARM1 in ferroptosis. f) Cell viability was assayed in the indicated LoVo cells treated with 2. 5 × 10^−6^ m RSL3 for 12 h (*n* = 5 independent experiments). g,h) Malondialdehyde (MDA) levels and relative lipid ROS were assayed in the indicated LoVo cells treated with 2. 5 × 10^−6^ m RSL3 for 12 h (*n* = 3 independent experiments). i) The indicated stable LoVo cells were subcutaneously injected into the mice, and RSL3 was injected intratumorally (100 mg kg^−1^, twice per week). Tumor volumes (*n* = 5) were calculated every 4 days, and the growth curve was drawn. j) Images of tumors from LoVo xenograft mice with altered treatments are shown, and the tumor weights (*n* = 5) of the subcutaneous xenografts were measured. k) Representative immunohistochemical images of CARM1, ACSL4, and Ki67 in tumor sections are shown. Scale bars, 20 µm. l,m) MDA levels and relative lipid ROS in tumor cells isolated from (j) were assayed (*n* = 5 independent experiments). The data shown represent the mean ± SD. In (d), comparisons were made by using the two‐tailed, unpaired Student's *t*‐test; All other comparisons were made by using one‐way ANOVA with Tukey's test; ^*^ *p* < 0.05, ^**^ *p* < 0.01, ^***^ *p* < 0.001; n.s., no significant difference.

To explore the molecular mechanisms underlying the ferroptosis‐suppressive effects of CRC mediated by CARM1, we hypothesized that CARM1 regulates ferroptosis by affecting the generation or scavenging of lipid peroxides. As previously confirmed, CARM1 inhibited ferroptosis in the presence of RSL3‐induced GPX4 deletion, and knockdown of CARM1 showed no significant difference in the protein levels of GPX4 or cellular divalent iron levels (Figure 1g, Figure S3a,b, Supporting Information). Therefore, we investigated whether CARM1 regulates ferroptosis by affecting PUFA metabolism, which has been reported to dictate ferroptosis sensitivity. Among the various membrane phospholipids, AA‐ and AdA‐containing phosphatidylethanolamine (PE) are considered primary targets for lipid peroxidation. PE‐AA and PE‐AdA can be incorporated into phospholipids and oxidized by lipoxygenases, which damage the membrane integrity and result in ferroptosis.^[^ ^26^, ^27^, ^28^ ^]^ Therefore, we analyzed all major PE species in control and CARM1‐KD LoVo cells and found that PUFAs, including AA‐ and AdA‐containing PE species, accumulated in CARM1‐KD cells (**Figure** **3**a–d). We identified 10 potential substrates in our mass spectrometry analysis of CARM1, including two PUFA enzymes (ACSL3 and ACSL4). To further define the substrate of CARM1 that moderates ferroptosis, cell viability assays were performed on shNC, shCARM1 LoVo, and HCT116 cells by knocking down ACSL3 or ACSL4 following treatment with RSL3. The results showed that ACSL4 but not ACSL3 knockdown rescued ferroptosis in shCARM1 cells (Figure 3f, Figure S3c, Supporting Information). Subsequently, we constructed stable CARM1 and ACSL4 double knockdown cell lines (Figure S3d, Supporting Information). CARM1 and ACSL4 double knockdown also reduced MDA and lipid peroxidation levels compared to those in CARM1‐KD cells (Figure 3g,h, Figure S3e,f, Supporting Information). Furthermore, fluorescence staining with JC‐1 dye and TEM demonstrated that ACSL4 knockdown reduced mitochondrial damage in shCARM1 cells (Figure S3g,h, Supporting Information).

Subsequently, we validated the role of ACSL4 in CARM1‐induced tumor growth in vivo. ShNC, shCARM1, CARM1, and ACSL4 double‐knockdown LoVo cells were subcutaneously transplanted into nude mice and all tumors were subjected to RSL3 treatment. The tumor growth curve and IHC results showed that ACSL4 knockdown accelerated the growth of shCARM1 tumors by increasing Ki‐67 levels (Figure 3i,k). Moreover, ACSL4 knockdown in shCARM1 tumors significantly increased tumor weight (Figure 3j) and reduced MDA and lipid peroxide levels (Figure 3l,m). Thus, we concluded that CARM1 inhibits ferroptosis via ACSL4.

### 2.4 CARM1 Directly Interacts with and Decreases ACSL4 Protein Levels in Colon Cancer Cells

**Figure 4:**
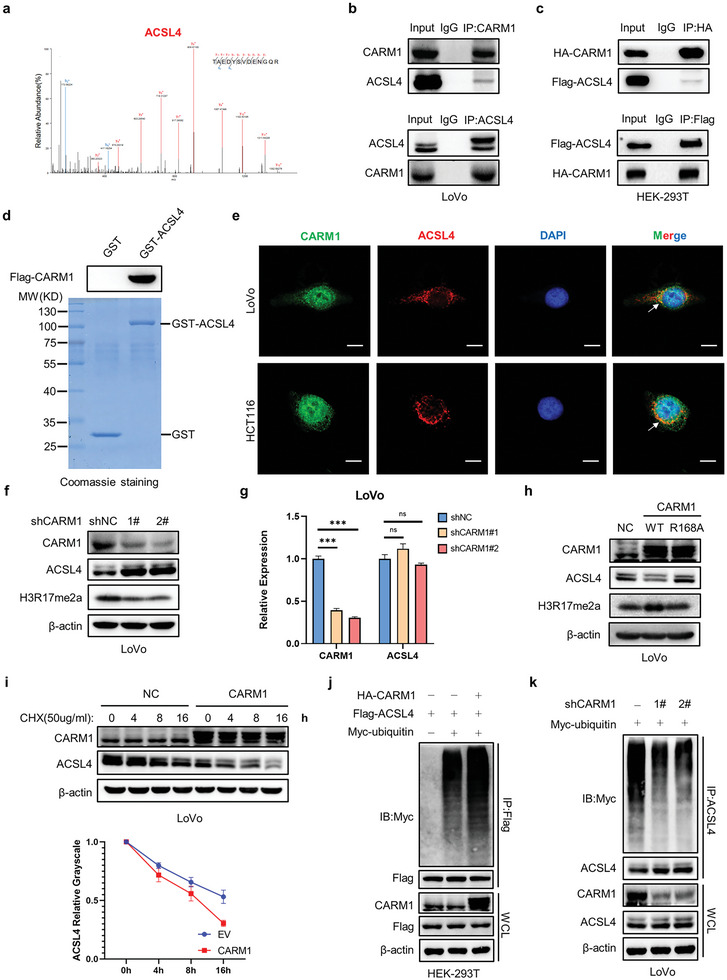
CARM1 directly interacts with and decreases ACSL4 protein levels in colon cancer cells. a) Mass spectrometry analysis identified ACSL4 in the binding protein pool of CARM1. b) Immunoprecipitation (IP) analyses were performed to examine the endogenous interaction between CARM1 and ACSL4 by using antibodies against CARM1 and ACSL4 in LoVo cells. c) IP analyses were performed to examine the exogenous interaction between CARM1 and ACSL4 by using antibodies against Flag and HA, respectively, in HEK293T cells. d) In vitro GST pull‐down assay to verify the binding of CARM1 and ACSL4. e) Immunofluorescence staining was performed to observe the colocalization of CARM1 (green) and ACSL4 (red) in LoVo and HCT116 cells. The nucleus is labeled via DAPI (blue). Scale bar, 20 µm. f) Western blot analysis of the indicated LoVo cells. Protein levels of CARM1, ACSL4, and H3R17me2a were assayed. g) Quantitative real‐time PCR (qPCR) analysis of CARM1 and ACSL4 mRNA levels in the indicated LoVo cells (*n* = 3 independent experiments). h) Western blot analysis of the indicated LoVo cells. Protein levels of CARM1, ACSL4, and H3R17me2a were assayed. i) Western blot analysis of vector‐ and CARM1‐overexpressing LoVo cells treated with 50 µg mL^−1^ cycloheximide for the indicated times. Quantitative analysis was conducted on ACSL4 levels at the indicated time points. j) IP with an anti‐Flag antibody and Western blotting with an anti‐Myc antibody were performed to detect the ubiquitination level of ACSL4. k) CARM1‐knockdown cells were transfected with the indicated plasmid and treated with MG132 (10 × 10^−6^ m) for 8 h. IP with an anti‐ACSL4 antibody and Western blot analysis of the ubiquitination of endogenous ACSL4 were performed. The data shown represent the mean ± SD. Comparisons were made by using one‐way ANOVA with Tukey's test; ^***^ *p* < 0.001; n.s., no significant difference.

As ACSL4 was detected by CARM1 mass spectrometry (**Figure** **4**a), we performed co‐immunoprecipitation (co‐IP) assays, and the results demonstrated an endogenous physical interaction between CARM1 and ACSL4 (Figure 4b). In addition, HA‐CARM1 and Flag‐ACSL4 plasmids were co‐transfected into HEK‐293T cells, and co‐IP was performed to verify the exogenous interaction; consequently, a consistent result was obtained (Figure 4c). Furthermore, an in vitro pull‐down assay confirmed the binding of CARM1 to GST‐ACSL4 (but not to GST alone) (Figure 4d). In addition, immunofluorescence was performed in LoVo and HCT116 cells to observe the colocalization of CARM1 and ACSL4 (Figure 4e, Figure S4a, Supporting Information). We also investigated the interactions between CARM1 and ACSL4. First, CARM1‐KD resulted in the accumulation of endogenous ACSL4, whereas changes in the transcript levels of ACSL4 were negligible (Figure 4f,g, Figure S4b,c, Supporting Information). To further explore whether CARM1 decreases ACSL4 protein expression, we transfected different CARM1 overexpression plasmids into CRC cells and found that overexpression of wild‐type (WT) CARM1, but not the CARM1 R168A mutant deficient in methyltransferase activity,^[^ ^29^ ^]^ significantly decreased ACSL4 protein levels (Figure 4h, Figure S4d, Supporting Information).

Considering that both proteasomes and lysosomes can mediate protein degradation, we treated cells with MG132, a reversible proteasome inhibitor, or chloroquine (CQ), an autophagy and Toll‐like receptor inhibitor, to indirectly evaluate protein synthesis efficiency. The results demonstrated that MG132 (but not CQ) reversed the degradation of ACSL4 protein in CARM1‐overexpressing cells (Figure S4e, Supporting Information). Next, we determined the half‐life of ACSL4 when CARM1 was overexpressed in LoVo and HCT116 cell lines. CHX (50 µg mL^−1^) was administered to each group of cells at the indicated time points and total lysates were collected to detect ACSL4 protein levels. The results showed that the overexpression of CARM1 promoted ACSL4 degradation in LoVo and HCT116 cells (Figure 4i, Figure S4f, Supporting Information). Based on these results, we concluded that CARM1 significantly accelerates endogenous ACSL4 degradation and shortens its half‐life. To determine whether CARM1 regulates ubiquitin‐mediated degradation of ACSL4 protein, we used a ubiquitination assay to detect the ubiquitylation level of endogenous ACSL4 protein. Ubiquitination‐based IP showed that the ubiquitylation level of ACSL4 protein was significantly decreased in CARM1‐knockdown LoVo cells, whereas overexpression of CARM1 significantly increased exogenous ACSL4 ubiquitylation in HEK293T cells (Figure 4j,k).

### 2.5 CARM1 Accelerates ACSL4 Protein Degradation by Methylating ACSL4 at R339

**Figure 5:**
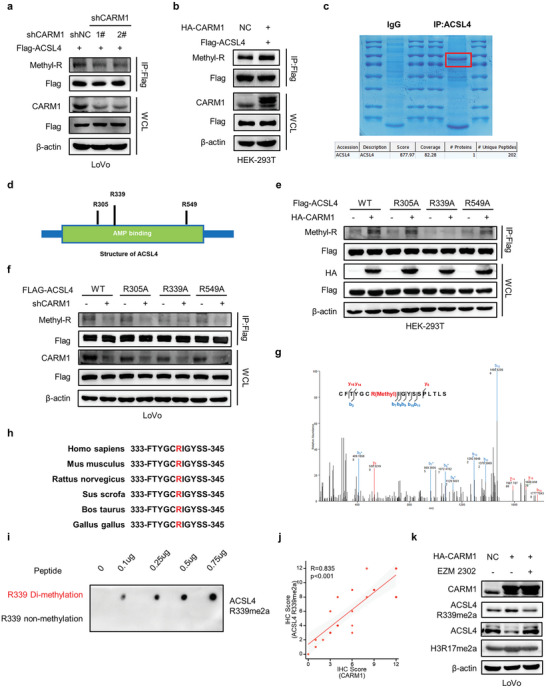
CARM1 methylates ACSL4 at R339. a,b) Co‐immunoprecipitation (Co‐IP) was performed to detect the methylation levels of ACSL4 with CARM1 attenuation (left) or upregulation (right). c) IP assay was performed for the enrichment of ACSL4 protein, staining was performed with Coomassie bright blue and verification was conducted by using mass spectrometry. d) Schematic diagram of ACSL4 structure and methylation sites. e) Co‐IP was performed to detect the methylation changes in WT ACSL4 and the R305A, R339A, and R549A mutants with CARM1 overexpression. f) Co‐IP was performed to detect the methylation changes in WT ACSL4 and the R305A, R339A, and R549A mutants with CARM1 attenuation. g) Secondary mass spectrometry result of one possible methylation residue at arginine 339. h) The ACSL4 R339 site amino acid in different species. i) Dot plot assay verifying the specificity of anti‐ACSL4 R339me2a using 0.1‐0.75 µg of different peptides. j) Correlation between CARM1 and ACSL4 R339Ame2a expression in colorectal cancer (CRC) tissues (*n* = 25) was determined by using the Spearman correlation coefficient test. All *p* and *R* values were calculated with Spearman's *r* test. k) Western blot analysis of vector‐ and CARM1‐overexpressing LoVo cells treated with DMSO or 10 × 10^−9^ m EZM2302 for 24 h. Protein levels of CARM1, ACSL4, ACSL4 R339me2a, and H3R17me2a were assayed.

Considering that CARM1 is a methyltransferase, we speculated that CARM1 accelerates ACSL4 protein degradation by methylating ACSL4. A quantified IP assay was used to confirm this hypothesis, and the results showed that the downregulation of CARM1 reduced the methylation of ACSL4 protein (**Figure** **5**a), whereas the overexpression of CARM1 was associated with the hypermethylation of ACSL4 protein (Figure 5b). EZM2302, a selective inhibitor of CARM1 activity, successfully blocked the methylation and degradation of ACSL4 protein upon CARM1 overexpression (Figure S5a, Supporting Information). Consistent with these results, EZM2302 decreased the ubiquitination level of the ACSL4 protein (Figure S5b, Supporting Information). These findings elucidated the relationship between CARM1‐mediated methylation and ubiquitylation.

To determine the specific binding domain between CARM1, ACSL4, and the CARM1‐dependent methylation residue of ACSL4, we established different truncated forms of ACSL4 based on the native domain of the protein (full length, 1–711 aa; fragment 1, 1–102 aa; fragment 2, 103–578 aa; fragment 3, 579–711 aa) to identify the potential protein region that interacts with CARM1 (Figure S5c, Supporting Information). The co‐IP assay demonstrated that fragment 2 (103–578 AA) of ACSL4 interacted with CARM1 in HEK‐293T cells (Figure S5d, Supporting Information). To define the methylation sites of ACSL4, IP was performed to enrich ACSL4 protein for mass spectrometry (Figure 5c). Three arginine residues were methylated (R305, R339, and R549), all of which were located in fragment 2 of ACSL4 (Figure 5d). Next, we constructed mutant plasmids with R to A at three sites and overexpressed them in HEK‐293T or LoVo cells, followed by detection of the methylation status of ACSL4. The results showed that only the mutation at R339 inhibited the methylation level changes caused by changes in CARM1 expression (regardless of overexpression or downregulation) (Figure 5e,f). Secondary mass spectrometry results suggested that ACSL4 was methylated at R339 (Figure 5g). Notably, the R339 site was highly conserved among various species (Figure 5h). Furthermore, we synthesized and purified an R339‐specific asymmetric dimethylation antibody (anti‐ACSL4 R339me2a) that specifically recognizes ACSL4 R339 asymmetric dimethylation by using a dot blot assay (Figure 5i). IHC of CRC tissues showed that high CARM1 expression was positively correlated with high ACSL4 R339me2a expression (Figure 5j). Interestingly, ACSL4 R339me2a expression was significantly negatively correlated with lipid ROS and MDA levels in CRC tumor tissues of patients (Figure S5e, Supporting Information). Furthermore, as expected, EZM2302 significantly inhibited ACSL4 ubiquitylation by abrogating R339 dimethylation (Figure 5k, Figure S5b,f, Supporting Information). In conclusion, our results demonstrated that CARM1 promotes ACSL4 protein degradation by methylating ACSL4 at R339.

### 2.6 ACSL4 R339 Methylation Promotes the Degradation of ACSL4 by RNF25

**Figure 6:**
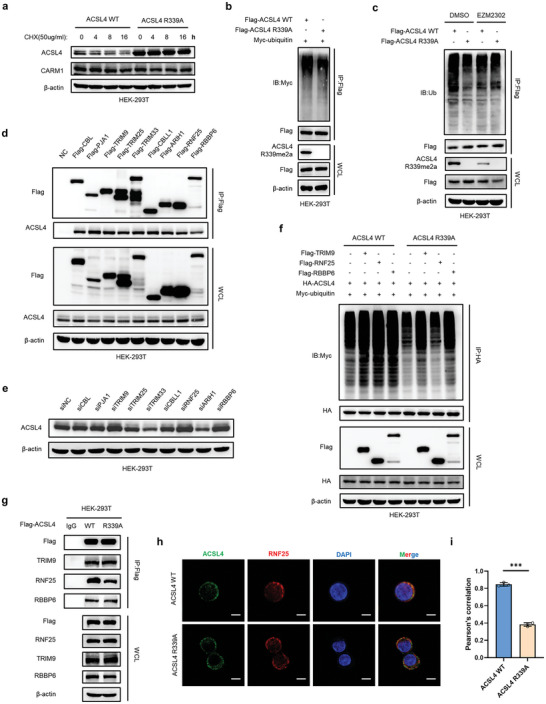
ACSL4 R339 methylation promotes the degradation of ACSL4 by RNF25. a) Western blot analysis of ACSL4 WT and ACSL4 R339A‐overexpressing stable HEK293T cells treated with 50 µg mL^−1^ cycloheximide for the indicated times. b) HEK293T cells transfected with the indicated plasmids and treated with 10 × 10^−6^ m MG132 for 8 h. Immunoprecipitation (IP) with anti‐Flag antibody and Western blotting with anti‐Myc antibody were performed to detect the ubiquitination level of ACSL4. c) HEK293T cells were treated as in (b) and treated with or without 10 × 10^−9^ m EZM2302 for 24 h. IP with an anti‐Flag antibody and Western blotting with an anti‐ubiquitin antibody were performed to detect the ubiquitination level of ACSL4. d) IP analyses were performed to examine the interaction between the indicated E3 ubiquitin ligase and endogenous ACSL4 using anti‐Flag antibodies in HEK293T cells. e) Western blot analysis of HEK293T cells transfected with the indicated siRNAs of E3 ubiquitin ligase. Protein levels of ACSL4 were assayed. f) HEK293T cells transfected with the indicated plasmids and treated with 10 × 10^−6^ m MG132 for 8 h. IP with anti‐HA antibody and Western blotting with anti‐Myc antibody were performed to detect the ubiquitination level of ACSL4. g) IP analyses were performed to detect the interaction changes between ACSL4 WT or ACSL4 R339A and the indicated E3 ubiquitin ligase. h) Immunofluorescence staining was performed to observe the colocalization changes of ACSL4 (green) and RNF25 (red) in ACSL4 WT and ACSL4 R339A LoVo cells. The nucleus is labeled by using DAPI (blue). Scale bar, 20 µm. i) Statistics of the colocalization of ACSL4 and RNF25, as indicated by Pearson's correlation (30 cells per sample). The data shown represent the mean ± SD. Comparisons were made by using two‐tailed, unpaired Student's *t*‐test; ^***^ *p* < 0.001.

To determine whether ACSL4 R339 methylation contributed to ACSL4 stability, ACSL4 WT or R339A mutant was stably expressed in HEK‐293T cells following CHX treatment. We found that the mutant R339A extended the half‐life of the ACSL4 protein and decreased ACSL4 ubiquitylation (**Figure** **6**a,b). In addition, the R339 mutation antagonized the decrease in ACSL4 ubiquitination induced by EZM2302 treatment (Figure 6c). These results indicated that CARM1‐mediated methylation of ACSL4 R339 promotes the degradation of ACSL4 by ubiquitination. Thus, we hypothesized that R339 methylation promotes binding of ubiquitin ligase to ACSL4. To verify this hypothesis, we screened all ubiquitin E3 ligases identified in our mass spectrometry analysis of the ACSL4 protein, including CBL, PJA1, TRIM9, TRIM25, TRIM33, CBLL1, ARIH1, RNF25, and RBBP6. Subsequently, we constructed plasmids encoding the aforementioned ligases and overexpressed them in HEK‐293T cells. IP results confirmed the physical interaction between ACSL4 and all ubiquitin ligases (Figure 6d, Figure S6a, Supporting Information). Next, small interfering RNAs (siRNAs) were used to knockdown these ubiquitin ligases in HEK‐293T cells, and we found that ACSL4 was upregulated in siTRIM9, siRNF25, and siRBBP6 cells (Figure 6e, Figure S6b, Supporting Information). Therefore, we speculated that TRIM9, RNF25, and RBBP6 are potential ubiquitin ligases that induced the degradation of ACSL4. Considering that the ACSL4 R339A mutant leads to poorer ubiquitylation, we performed a ubiquitination assay in which TRIM9, RNF25, or RBBP6 were co‐transfected with the ACSL4 WT or R339A mutant in HEK‐293T cells. As expected, TRIM9, RNF25, and RBBP6 enhanced the ubiquitylation of ACSL4 WT, whereas only the R339A mutant exhibited a negligible change in ubiquitylation when RNF25 was overexpressed (Figure 6f). To verify whether R339 plays a role in the binding of RNF25 and ACSL4, a co‐IP assay enriched with ACSL4 WT and ACSL4 R339A mutant proteins in HEK‐293T cells was performed. The R339A mutant decreased the binding of RNF25 to ACSL4 (Figure 6g). Similar results were observed for IF (Figure 6h,i).

As RNF25 was responsible for the ubiquitylation of methylated ACSL4 and its subsequent degradation, we further explored the types of RNF25‐mediated ACSL4 ubiquitylation. The results showed that RNF25 mediated ACSL4 K48‐linked polyubiquitination rather than K63‐linked polyubiquitination (Figure S6c,d, Supporting Information). Furthermore, the expression of ACSL4 was significantly negatively correlated with RNF25 protein expression in CRC tumor tissues of patients (Figure S6e–g, Supporting Information). These results demonstrated that ACSL4 R339 methylation promotes the ACSL4 degradation and is indispensable for the binding of RNF25 and ACSL4.

### 2.7 RNF25 Induces Ferroptosis Resistance by Cooperating with CARM1

**Figure 7:**
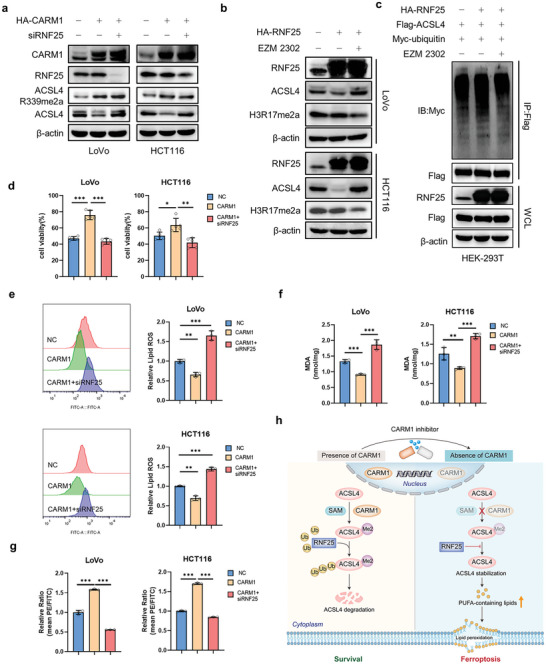
RNF25 knockdown inhibits CARM1‐induced ferroptosis resistance. a) Western blot analysis of LoVo and HCT116 cells transfected with the indicated plasmid and siRNAs. Protein levels of CARM1, RNF25, ACSL4 and ACSL4 R339me2a were assayed. b) Western blot analysis of vector‐ and RNF25‐overexpressing LoVo and HCT116 cells treated with DMSO or 10 × 10^−9^ m EZM2302 for 24 h. Protein levels of RNF25, ACSL4, and H3R17me2a were assayed. c) HEK293T cells transfected with the indicated plasmids and treated with or without 10 × 10^−9^ m EZM2302 for 24 h. Immunoprecipitation (IP) with an anti‐Flag antibody and Western blotting with an anti‐Myc antibody were performed to detect the ubiquitination level of ACSL4. d) Cell viability was assayed in the indicated LoVo and HCT116 cells as (a) treated with 2. 5 × 10^−6^ m RSL3 for 12 h (*n* = 5 independent experiments). e,f) Malondialdehyde (MDA) levels and relative lipid ROS were assayed in the indicated LoVo and HCT116 cells treated with 2. 5 × 10^−6^ m RSL3 for 12 h (*n* = 3 independent experiments). g) Mitochondrial membrane potential was detected for the same cells as (e) by using fluorescence staining of mitochondria with JC‐1 dye (*n* = 3 independent experiments). h) Schematic diagram of our hypothesis about this project. The data shown represent the mean ± SD. Comparisons were made by using one‐way ANOVA with Tukey's test; ^*^ *p* < 0.05, ^**^ *p* < 0.01, ^***^ *p* < 0.001.

In this study, we explored whether RNF25 induces ferroptosis resistance by cooperating with CARM1. Indeed, knockdown of RNF25 rescued the ACSL4 protein and demonstrated no significant difference in ACSL4 R339 dimethylation with CARM1 overexpression (**Figure** **7**a). Interestingly, RNF25‐mediated ubiquitylation and the subsequent degradation of ACSL4 were inhibited by EZM2302 (Figure 7b,c). Cell viability results showed that RNF25 knockdown significantly increased ferroptosis in CARM1‐overexpressing CRC cells (Figure 7d). The same conclusion was drawn from the results of MDA and lipid ROS levels (Figure 7e,f). JC‐1 dye assay showed that RNF25 knockdown promoted mitochondrial damage (Figure 7g). Overall, these data suggested that RNF25 knockdown aggravated ferroptosis by inhibiting CARM1‐induced ferroptosis resistance (Figure 7h).

### 2.8 Inhibition of Methylation at R339 of ACSL4 Aggravates ACSL4‐Induced Ferroptosis In Vitro and In Vivo

We have shown that CARM1 methylates ACSL4 at R339, and that this residue may influence the stability of ACSL4. To further explore the role of ACSL4 R339 methylation in ferroptosis, we generated ACSL4 knockout HCT116 and LoVo cell lines using the CRISPR/Cas9 system and subsequently stably expressed WT ACSL4 and a methylation‐deficient variant of ACSL4 R339A in ACSL4 knockout cell lines (Figure S7a, Supporting Information). After treatment with MG132, the ACSL4 protein levels were similar in ACSL4 WT and ACSL4 R339A cell lines (Figure S7b, Supporting Information). As shown in Figure S7c (Supporting Information), ACSL4 R339A was more sensitive to RSL3 treatment than ACSL4 WT (as expected). Moreover, the R339 mutation in ACSL4 elicited higher MDA and lipid peroxide levels than the control (Figure S7d,e, Supporting Information). Consistently, TEM and JC‐1 staining demonstrated that ACSL4 R339A resulted in severely shrunken mitochondria and increased membrane density (Figure S7f,g, Supporting Information).

To further evaluate the critical roles of ACSL4 methylation in ferroptosis in colon cancer in vivo, LoVo‐ACSL4‐KO, LoVo‐ACSL4‐WT, and LoVo‐ACSL4‐R339A cells were used to construct a mouse xenograft model. The tumor volume was measured, and a growth curve was plotted. The results showed that the R to A mutation at R339 inhibited tumor proliferation (Figure S7h, Supporting Information). Consistently, tumors from ACSL4 R339A mice were lighter, accompanied by increased MDA and lipid peroxidation levels (Figure S7i–k, Supporting Information). In conclusion, we demonstrated that the methylation‐defective mutant, R339A, of ACSL4 significantly improved ACSL4‐induced ferroptosis in vitro and in vivo.

### 2.9 EZM2302 Enhances the Efficacy of Immunotherapy by Promoting Ferroptosis

**Figure 8:**
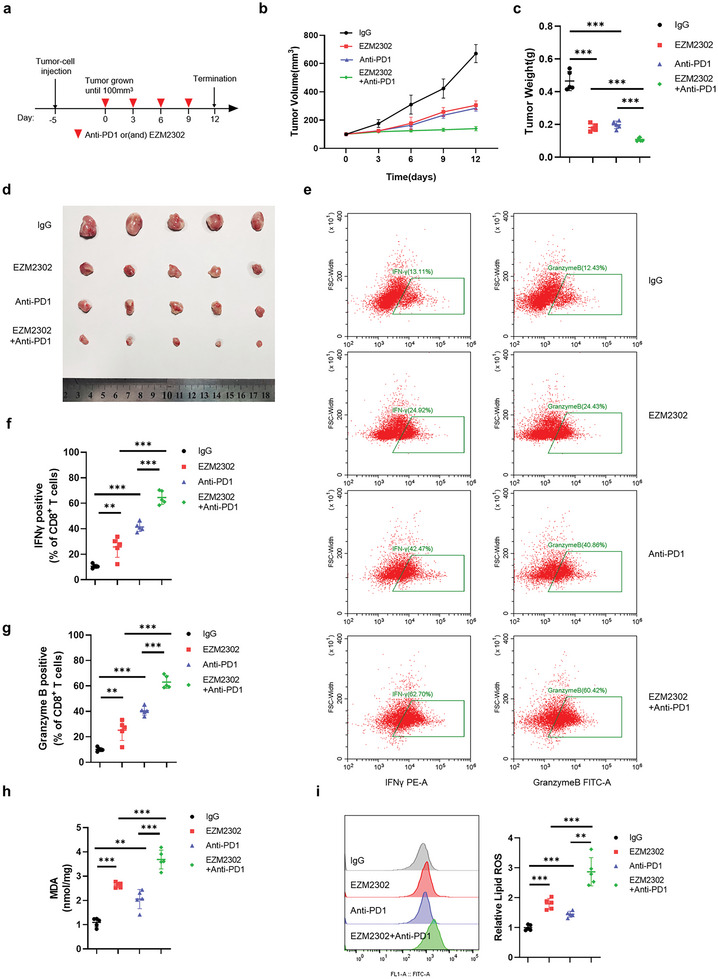
EZM2302 strengthens the efficacy of immunotherapy by promoting ferroptosis. a) Treatment protocol for MC38 xenografts in C57 mice following treatment with mouse anti‐PD1 antibody and EZM2302. b) MC38 cells were subcutaneously injected into the mice, and tumors were treated with mouse anti‐PD1 antibody and EZM2302. After tumors grew to 100 mm^3^, tumor volumes (*n* = 5) were calculated every 3 days, and the growth curve was drawn. c,d) Images of tumor size in different groups are shown, and the tumor weights (*n* = 5) of the subcutaneous xenografts were measured. e) Representative dot plot of mouse CD8^+^ T cells examined for the expression of interferon γ (IFN‐γ) (left) and granzyme B (GzmB) (right) after the indicated treatments. The proportions of cells with IFN‐γ or GzmB expression are shown on the left (*n* = 5 independent experiments). f,g) Representative contour plots of human peripheral CD8^+^ T cells examined for the expression of IFN‐γ (middle left) and granzyme B (GzmB) (bottom left) after the indicated treatments. h,i) Malondialdehyde (MDA) levels and relative lipid ROS in tumor cells isolated from (d) were assayed (*n* = 5 independent experiments). The data shown represent the mean ± SD. Comparisons were made by using one‐way ANOVA with Tukey's test; ^**^ *p* < 0.01, ^***^ *p* < 0.001.

Studies have shown that inactivation of CARM1 could sensitize tumors to T‐cell dependent antitumor immunity and that interferon γ (IFN‐γ), which is derived from tumor‐infiltrating CD8^+^ T cells, could trigger tumor cell ferroptosis.^[^ ^24^, ^30^, ^31^ ^]^ Considering that our study showed that EZM2302 stabilized ACSL4 by inhibiting CARM1, we proposed that EZM2302 sensitizes colon cancer cells to anti‐PD‐1 immunotherapy by promoting ferroptosis, whereas anti‐PD1 enhances EZM2302‐induced tumor cell ferroptosis. To verify this hypothesis, we established a mouse xenograft model by injecting MC38 cells into C57 mice. Five days after MC38 cell injection, EZM2302 and PD‐1 blockade or IgG was administered every 3 days (**Figure** **8**a). The results suggested that EZM2302 combined with anti‐PD‐1 significantly reduced tumor volume and weight, and delayed tumor growth compared to the use of anti‐PD‐1 alone (Figure 8b–d). Similar to previous studies, anti‐PD‐1 therapy greatly enhanced tumor infiltration, including increased accumulation of T cells expressing the effector molecules granzyme B and IFN‐γ (Figure 8e–g). Notably, administration of EZM2302 resulted in similar results to those of the anti‐PD1 antibody. Moreover, we found that the combined application of EZM2302 and anti‐PD1 further improved CD8^+^ T‐cell activation, indicating that EZM2302 promotes the effect of immunotherapy. In addition, we measured MDA and lipid peroxidation levels to evaluate ferroptosis in the tumors. The results showed that both EZM2302 and anti‐PD1 alone increased MDA and lipid peroxidation levels; however, the combined use of EZM2302 and anti‐PD1 enhanced this effect (Figure 8h,i). Overall, we concluded that the combination of EZM2302 and anti‐PD1 strengthens the efficacy of immunotherapy and ferroptosis in vivo.

## 3 Discussion

Studies have demonstrated the importance of ferroptosis in tumor progression; however, the molecular triggers of ferroptosis remain unclear.^[^ ^8^, ^11^ ^]^ Our data confirmed that CARM1 inhibited ferroptosis by directly interacting with and methylating ACSL4 at R339. Subsequently, methylated ACSL4 interacts with RNF25 and is degraded by RNF25‐induced ubiquitylation, which decreases lipid peroxidation products and slows the progression of ferroptosis. The phenomenon of methylation‐ubiquitylation cross‐talk may contribute to stabilizing the intracellular environment and provide new clues regarding the combination of drugs that are used in cancer therapy.

Along with phosphorylation, acetylation, and ubiquitylation, methylation is an important PTM of proteins and has been extensively studied in recent years.^[^ ^32^, ^33^ ^]^ Unlike previous studies on the methylation of histone proteins involved in the maintenance of chromosomal stability and transcription, the methylation of lysine and arginine residues on non‐histone proteins has been shown to be a common modification of proteins and has been reported to mediate multiple cellular processes.^[^ ^23^, ^34^, ^35^ ^]^ The cross‐talk between methylation and other modifications regulates protein stability, activity, and intracellular location; therefore, these transmethylases are considered potential targets for combined therapy. For example, SETDB1 has been reported to promote the activity of the PI3K/AKT signaling pathway by methylating AKT at K64 and K140/142; thus, mithramycin‐targeting SETDB1 could effectively improve sensitivity to cetuximab treatment.^[^ ^36^ ^]^

CARM1 is a histone methyltransferase that methylates histone H3 at ‘Arg‐17′ (H3R17me) and plays a crucial role in nonhistone protein methylation.^[^ ^37^ ^]^ CARM1 has been reported to dimethylate LSD1 at R838, resulting in the deubiquitination and stabilization of LSD1, and promoting the invasion and metastasis of breast cancer cells.^[^ ^38^ ^]^ In another study, CARM1 reprogrammed cancer metabolism by methylating PKM2 at R445, R447, and R455, with changes in its enzyme activity.^[^ ^23^ ^]^ In this study, we identified ACSL4 as a substrate of CARM1 and a specific methylated amino acid residue. The discovery of these new substrates has broadened the understanding of the mechanism of CARM1 in tumor progression.

In ferroptosis, the accumulation of lipid peroxidation products and violent Fenton reaction are major causes of cell death. Several studies have focused on GPX4‐mediated elimination of lipid peroxides.^[^ ^16^, ^39^ ^]^ However, PUFA‐containing lipid biosynthesis provides the raw material for the Fenton reaction, thus leading to ferroptosis. ACSL4 has been widely reported to be a vital proferroptosis gene.^[^ ^17^, ^18^, ^40^ ^]^ ACSL4 is a metabolic enzyme that plays a key role in the biosynthesis of PUFA‐containing lipids.^[^ ^41^ ^]^ ACSL4 knockout prevents GPX4 deletion‐induced ferroptosis by altering the lipid composition of cells.^[^ ^17^ ^]^ In addition, the Thr328 site of ACSL4 is activated by PKCβII phosphorylation, which increases the activity of ACSL4 and aggravates ferroptosis.^[^ ^15^ ^]^ Although the abovementioned studies have elucidated the function of ACSL4 (to a certain extent), the degradation, localization, and protein interactions of ACSL4 remain unclear. Our study elucidated the function of methylated ACSL4 protein and confirmed that ACSL4 R339 methylation contributes to the binding of ACSL4 and RNF25. Studies have shown that lysine or arginine methylation can significantly alter the hydration energy and hydrogen bonding potential of these side chains.^[^ ^42^, ^43^, ^44^ ^]^ The methylation of ACSL4 may alter the intermolecular forces between ACSL4 and RNF25, such as hydrogen bonds, thereby strengthening the ACSL4‐RNF25 interaction. This scenario explains why R339‐methylated ACSL4 was more closely integrated with RNF25 and more easily degraded via RNF25‐induced ubiquitination. However, the complete structure of ACSL4 is not yet fully understood and we cannot provide reliable structural evidence. Additionally, the ubiquitination site of RNF25‐mediated ACSL4 degradation was not well defined in this study. Moreover, studies on other E3 ubiquitin ligases (such as TRIM9 and RBBP6, which we found can also downregulate ACSL4) are needed to further elucidate this mechanism. These data can further enhance our understanding of the role of ACSL4 in ferroptosis.

ROS have been reported to be cleared by CARM1 by methylating RPIA and enhancing NADPH generation.^[^ ^45^ ^]^ Notably, in our study, both ROS and lipid ROS were eliminated by CARM1, increasing the possibility that CARM1 functions in ferroptosis. We subsequently demonstrated that CARM1 moderates ferroptosis, thus facilitating tumor progression. As previously mentioned, ferroptosis is regulated by multiple mechanisms. In addition to GPX4‐GSH‐axis‐mediated ROS scavenging, the transport and transformation of divalent iron and metabolism of PUFAs are involved in the production and clearance of lipid ROS.^[^ ^28^, ^46^, ^47^ ^]^ Liu and Cao confirmed that CARM1 is associated with fatty acid metabolism.^[^ ^48^, ^49^ ^]^ Using mass spectrometry, we identified ACSL4 as a downstream molecule of CARM1 that mediates ferroptosis. In view of the fact that inhibition of CARM1 induces beneficial antitumor activity in cytotoxic T cells and tumor cells, the combination of the CARM1 inhibitor EZM2302 and anti‐PD‐1 has been shown to be mutually reinforcing in strengthening ferroptosis and promoting immunotherapeutic efficacy, and the combined effect is significantly better than that of each molecule alone.

In summary, we found and provided evidence that CARM1 inhibited ferroptosis, which is closely related to ACSL4 R339 methylation, and that its inhibitor suppressed tumor progression when combined with anti‐PD‐1. These results provide new ideas and directions for the development of novel clinical therapeutic targets for ferroptosis and related drugs (either alone or in combination).

## 4 Experimental Section

### Antibodies and Reagents

Antibodies against CARM1 (#3379), β‐actin (#3700), Flag‐tag (#14 793), Myc‐tag (#2276), HA‐tag (#3724), TRIM33 (#90 051), and asymmetric di‐methyl arginine motif (adme‐R) (#13 522) were purchased from Cell Signaling Technology. Primary antibodies against ACSL4 (22401‐1‐AP) were purchased from Proteintech. Antibodies against ubiquitin (ab134953), TRIM9 (ab300515), TRIM25 (ab167154), TRIM33 (ab300146), RNF25 (ab140514), and Ki67 (ab16667) were purchased from Abcam. CBL (SAB4503444) and PJA1 (HPA000595) were purchased from Sigma‒Aldrich. Additionally, CBLL1 (sc‐517157), ARIH1 (sc‐390763), and RNF25 (sc‐398749) were purchased from Santa Cruz Biotechnology. H3R17me2a (49‐1021) was purchased from Thermo Fisher Scientific. The asymmetric dimethyl‐ACSL4 (Arg339) rabbit polyclonal antibody was purchased from AtaGenix, China (http://www.atagenix.com). Moreover, the antibodies and dyes that were used for flow cytometry, including the Zombie NIR Fixable Viability Kit (#423 105), Percp5, 5‐CD45 (#103 130), BV650‐CD8 (#100 741), FITC‐Granzyme B (#372 205), and PE‐IFN‐γ (#505 807), were obtained from BioLegend. The secondary antibodies DyLight 549 goat anti‐rabbit IgG (A23320), DyLight 488 goat anti‐mouse IgG (A23210), DyLight 488 goat anti‐rabbit IgG (A23220), and DyLight 549 goat anti‐mouse IgG (A23310) were purchased from Abbkine.

The reagents for RSL3 (HY‐100218A), erastin (HY‐15763), Fer‐1 (HY‐100579), Z‐VAD‐FMK (HY‐16658B), NEC (HY‐15760), NAC (HY‐B0215), CHX (HY‐12320), MG132 (HY‐13259), CQ (HY‐17589A), and EZM2302 (HY‐111109) were purchased from MedChem Express.

### Cell Culture

The colon cancer cell lines LoVo, HCT116, and MC38, as well as the human embryonic kidney cell line HEK293T, were obtained from the American Type Culture Collection. All the cell lines were cultured at 37 °C in an incubator with 5% CO_2_. The media used for LoVo, HCT116, and HEK293T cells were supplemented with DMEM (GIBICO, #12 800), 10% FBS (HyClone, SV30160. 03), and 1% penicillin/streptomycin, respectively. MC38 cells were cultured in RPMI‐1640 (#31 800, GIBICO) with 10% FBS and 1% penicillin/streptomycin.

### siRNAs and Plasmids

siRNAs targeting CARM1, CBL, PJA1, TRIM9, TRIM25, TRIM33, CBLL1, RNF25, ARIH1, and RBBP6 were designed and synthesized by Guangzhou RiboBio Co., Ltd. The sequences are listed in Table S1 (Supporting Information). Expression vectors encoding pcDNA3.1‐HA‐CARM1, pcDNA3.1‐Flag‐ACSL4, pcDNA3.1‐Myc‐ubiquitin, pcDNA3.1‐Flag‐PJA1, pcDNA3.1‐Flag‐TRIM9, pcDNA3.1‐Flag‐TRIM25, pcDNA3.1‐Flag‐TRIM33, pcDNA3.1‐Flag‐CBLL1, pcDNA3.1‐Flag‐RNF25, and pcDNA3.1‐Flag‐ARIH1 were purchased from AUGCT (http://www.augct.com). Additionally, pcDNA3.1‐Flag‐CBL, pcDNA3.1‐Flag‐PJA1, pcDNA3.1‐Flag‐RBBP6, and pLVX‐ACSL4 plasmids were constructed by inserting the indicated DNAs into the indicated vector. The CARM1 and ACSL4 mutants were generated using the Mut Express II Fast Mutagenesis Kit V2 (C214‐01, Vazyme). Moreover, ACSL4‐truncated and deletion mutants were established using the pcDNA3.1‐Flag‐ACSL4 plasmid. The primers used for the ACSL4 site and deletion mutants are listed in Table S2 (Supporting Information). All plasmids were confirmed by sequencing.

### Establishment of Stable Cell Lines

The lentiviral shRNA vector pLKO, 1‐shCARM1 or ACSL4, MD2‐G, and the PPAX three‐pack system was used for obtaining silencing‐expression viruses. The sequences are listed in Table S1 (Supporting Information). The pLVX‐indicated genes, MD2‐G, and PPAX three‐pack system were used to generate a high‐expression virus. All viruses were transfected into the specified cells. After 12 h, the medium was replaced with a fresh complete medium. After 48 h, 1 µg mL^−1^ puromycin was used to obtain stable cell lines. Additionally, CRISPR/Cas9 technology was used to knock out ACSL4, and sgRNA was inserted into the empty backbone of lenti‐CRISPR v2. The sgACSL4 sequences are listed in Table S1 (Supporting Information). Lipofectamine 3000 transfection reagent (#11 668 030, Thermo Fisher Scientific) was used for transfection following the manufacturer's instructions. After 48 h, the cells were selected with 1 µg mL^−1^ puromycin for 3 days. Individual clones of colon cancer cell lines expressing ACSL4 were confirmed using Western blotting.

### Cell Viability Assay

Cell viability was assessed using the Cell Counting Kit‐8 (CCK‐8; HY‐K0301, MedChemExpress). LoVo and HCT116 cells were seeded in 96‐well plates at a density of 5 × 10^4^ cells per well and treated with test compounds. At the indicated times, 10 µL of the CCK‐8 solution was added to each well of the plate. After incubation for 5 h, the absorbance of the plate was measured at 450 nm. The cell viability under the indicated conditions is shown as a percentage relative to that of the negative control.

### Measurement of ROS and Lipid ROS Levels

The ROS and lipid ROS levels in the cell lines were measured using flow cytometry. The indicated cell lines were seeded in six‐well plates at a density of 3 × 10^5^ cells per well before the experiment. On the second day, the cells were collected, incubated with PBS containing 25 × 10^−6^ m DCFH‐DA (35 845, Sigma‒Aldrich) or 5 × 10^−6^ m C11‐BODIPY 581/591 (RM02821, ABclonal) at 37 °C for 30 min, resuspended in 500 µL of fresh PBS, and analyzed using a flow cytometer (FACSuite, BD Biosciences) equipped with a 488 nm laser for excitation. Measurement of lipid ROS levels in tissues required the production of single‐cell tissue suspensions using collagenase IV (C4‐BIOC, Sigma‒Aldrich) and hyaluronidase (Solarbio, H8030). Single‐cell suspensions were incubated with the Zombie NIR Fixable Viability Kit and 5 × 10^−6^ m C11‐BODIPY 581/591, as described previously. Dead cells were excluded by setting the gate with the Zombie Viability Dye, and the lipid ROS levels of viable cells were detected. Data were collected from the FITC and APC‐Cy7 channels, and the mean fluorescence was analyzed using FlowJo Version 10. 8 software.

### MDA Assay

After the tissues or cells were homogenized or lysed, MDA levels in the supernatants were measured using a lipid peroxidation MDA assay kit (S0131, Beyotime) according to the manufacturer's instructions.

### Labile Iron Pool Measurement

The indicated cell lines were seeded in six‐well plates at a density of 1 × 10^6^ cells per well before the experiment. After the cells were treated with the indicated reagents, they were collected and the divalent iron content was analyzed using a divalent iron detection kit according to the manufacturer's protocol (ab83366, Abcam).

### TEM

The indicated samples were treated with RSL3 (2.5 × 10^−6^ m) for 12 h. Cells were fixed with 2.5% glutaraldehyde in 0.1 × 10^−3^ m phosphate buffer and subsequently treated with 1% OsO4 for 2 h. After dehydration, the cells were embedded in epoxy resin. Ultrathin sections were prepared using an ultramicrotome, stained with lead citrate and uranyl acetate, and the distribution of mitochondria was observed using a transmission electron microscope.

### Mitochondrial Membrane Potential Assay

Mitochondrial membrane potential was assessed using the fluorescent dye JC‐1 (C2006, Beyotime) according to the manufacturer's instructions. The samples were analyzed using flow cytometry. Data were collected from the FITC and PE channels, and the mean fluorescence was analyzed using FlowJo Version 10. 8 software.

### Immunofluorescence

The cells were seeded onto coverslips. The cells were fixed in 4% paraformaldehyde for 15 min at 25 °C. Subsequently, the fixed cells were permeabilized in 0.1% Triton X‐100 for 5 min and incubated with primary antibodies overnight at 4 °C. After washing the coverslips thrice with PBS, the cells were incubated with secondary antibodies for 1 h and DAPI for 15 min at room temperature. The cells were imaged using a multiphoton confocal laser scanning microscope (Olympus FLUOVIEW FV1000). Images were processed using ImageJ software.

### IHC

Paired carcinoma and adjacent tissue specimens were collected from patients diagnosed with CRC. IHC analysis of paraffin‐embedded CRC specimens was performed according to the manufacturer's protocol. IHC staining was evaluated by two independent gastrointestinal pathologists. IHC scoring was based on the extent and intensity of staining. The percentage of positively stained cells was scored as follows, 0 (<10%), 1 (10–25%), 2 (26–50%), 3 (51–75%), and 4 (>75%). The staining intensity was scored as 0 (negative staining), 1 (mild staining), 2 (moderate staining), or 3 (strong staining). The IHC score was calculated by multiplying the percentage of positively stained cells with the staining intensity, which ranged from 0–12. Specimens with IHC scores ≥ 6 were defined as having high expression and those with scores <6 were defined as having low expression.

### GST Pull‐Down Assay

The proteins for CARM1 (OriGene, TP317483) and ACSL4 (H00002182‐P01, Abnova) were commercially available. The CARM1 protein was incubated with GST or GST‐ACSL4 fusion proteins bound to glutathione Sepharose beads at 4 °C overnight. GST and GST fusion proteins were boiled, subjected to SDS‐PAGE, and verified by Coomassie Blue staining. Binding proteins were detected by Western blotting.

### Western Blotting and IP

Cells were washed three times with cold PBS and lysed with NP‐40 buffer supplemented with a cocktail protease inhibitor (HY‐K0010, MedChemExpress) for 30 min at 4 °C. The protein concentrations were measured using a bicinchoninic acid assay kit (#23 225, Thermo Fisher Scientific). The proteins were then electrophoresed in SDS‐PAGE gels and transferred onto polyvinylidene difluoride membranes. After blocking with 5% non‐fat milk, the membranes were incubated with primary antibodies overnight at 4 °C. After incubation with 1:5000 horseradish peroxidase‐linked secondary antibodies at 25 °C for 2 h, the membranes were visualized using an efficient chemiluminescence kit (#34 096, Thermo Fisher Scientific).

For IP, proteins were extracted as described for the Western blot assay. After centrifugation, the supernatant was incubated with magnetic beads (HY‐K0205; MCE) overnight at 4 °C. The proteins were eluted from the magnetic beads and collected for subsequent experiments.

### qPCR

RNA was extracted using TRIzol (Takara, Japan) according to the manufacturer's instructions and reverse‐transcribed using the HiScript III 1st Strand cDNA Synthesis Kit (R312‐02, Vazyme). qPCR was performed using the ChamQ Universal SYBR qPCR Master Mix (Q711‐02, Vazyme) on an ABI 7300 QuantStudio3 PCR system. The primer sequences used for qPCR are listed in Table S3.

### Mass Spectrometry Analysis

CARM1 and ACSL4 protein samples were enriched using an IP assay and separated using SDS‐PAGE. Gel bands of interest were sent to the National Protein Science Facility at the School of Life Sciences, Tsinghua University. The gel was reduced with 5 × 10^−3^ m DTT and alkylated with 11 × 10^−3^ m iodoacetamide. The alkylated gel was then digested with trypsin in 50 × 10^−3^ m ammonium bicarbonate at 37 °C overnight. The peptides were extracted twice with 0.1% trifluoroacetic acid in 50% acetonitrile aqueous solution, redissolved in 0.1% trifluoroacetic acid, and analyzed using an Orbitrap Fusion mass spectrometer. The Proteome Discoverer node ptmRS was used to map the protein peptides and potential protein methylation (K/R) sites.

### LC‒MS

LC‒MS was supported by Metabo‐Profile Biotechnology Co., Ltd. (Shanghai).

### Animal Experiments

Female BALB/c nude mice and C57 mice (6–7 weeks old) were used to generate the xenograft models. CRC cells were collected and washed twice with PBS. The cells were mixed with Matrigel at a 1:1 ratio and subcutaneously injected with 2 × 10^5^/100 µL CRC cells. In some trials, RSL3 (100 mg kg^−1^) was intratumorally injected twice per week and Fer‐1 (2 µg mg^−1^) was intraperitoneally injected daily for 14 days. In xenograft models generated in C57 mice, anti‐PD1 (100 µg per injection) was intraperitoneally injected and vehicle EZM2302 (150 mg kg^−1^) was administered orally every 3 days. After 17 or 20 days, the mice were sacrificed and xenografts were collected for subsequent tests.

### Patients and Subject Details

This study was approved by the Tongji Hospital Ethics Committee (TJ‐IRB20220723). The clinical specimens used in this study were obtained from the Department of Gastrointestinal Surgery, Tongji Hospital. Demographic information, including age and sex, is presented in Table S4.

### Statistical Analysis

All statistical analyses were performed using GraphPad Prism 8. 0 (GraphPad, San Diego, CA). Data are represented as mean ± SD. The sample size (*n*) for each statistical analysis is shown in the figure legends. Survival curves were constructed using Kaplan–Meier analysis with the log‐rank test. Pearson's correlation analysis and Spearman's test were performed. All other comparisons were made using Student's two‐tailed *t*‐test and one‐way ANOVA with Tukey's test to determine the statistical significance of differences between groups. The statistical significance was set at *p* < 0.05; ^*^ *p* < 0.05, ^**^ *p* < 0.01, and ^***^ *p* < 0.001.

## Conflict of Interest

The authors declare no conflict of interest.

## Author Contributions

XL conceived and designed the experiments. SF performed most of the experiments. ZR, JZ and XS performed animal experiments. KW, YC and HL collected biological samples and analyzed the data. CZ supports all mass‐spec analysis. Other authors give suggestions for many experiments. SF and XL organized and analyzed the data and wrote the manuscript.

## Supporting information

Supporting Information

## Data Availability

The data that support the findings of this study are available from the corresponding author upon reasonable request.
