# Correction to “Inhibition of CARM1‐Mediated Methylation of ACSL4 Promotes Ferroptosis in Colorectal Cancer”

**DOI:** 10.1002/advs.75696

**Published:** 2026-05-13

S. Feng, Z. Rao, J. Zhang, X. She, Y. Chen, K. Wan, H. Li, C. Zhao, Y. Feng, G. Wang, J. Hu, X. Luo, “Inhibition of CARM1‐Mediated Methylation of ACSL4 Promotes Ferroptosis in Colorectal Cancer,” *Advanced Science* (Weinh) 10, no. 36 (2023): e2303484, https://doi.org/10.1002/advs.202303484.

An error was identified in the ACSL4 ubiquitination band in Figure 4K, where the band was inadvertently misplaced during figure preparation. The corrected figure has now been provided, and the corresponding raw images are included. This correction does not affect the results or conclusions of the study.


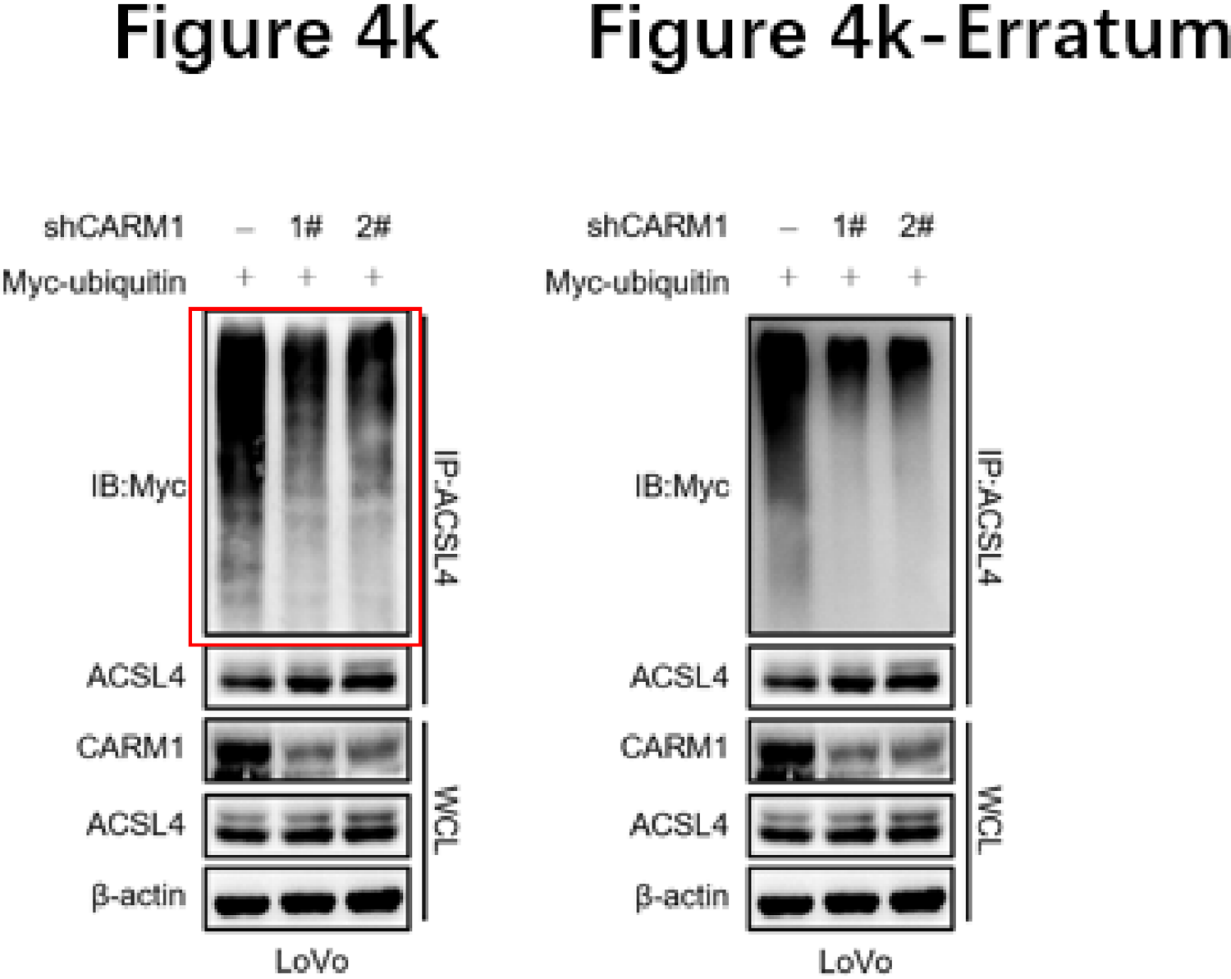


We apologize for this error.

