# Supplementary material for: Inhibition of CARM1‐Mediated Methylation of ACSL4 Promotes Ferroptosis in Colorectal Cancer
Source: Adv Sci (Weinh). 2023 Nov 9;10(36):2303484. doi: 10.1002/advs.202303484 (PMC10754121; doi:10.1002/advs.202303484)
Supplement: Supplementary file 1 — Supporting Information [file ADVS-10-2303484-s001.pdf]

ADVANCED SCIENCE
Open Access

Supporting Information

for *Adv. Sci.*, DOI 10.1002/advs.202303484

Inhibition of CARM1-Mediated Methylation of ACSL4 Promotes Ferroptosis in Colorectal Cancer

*Shengjie Feng, Zejun Rao, Jiakun Zhang, Xiaowei She, Yaqi Chen, Kairui Wan, Haijie Li, Chongchong Zhao, Yongdong Feng, Guihua Wang, Junbo Hu and Xuelai Luo**

Supplementary information

Inhibition of CARM1-mediated methylation of ACSL4 promotes ferroptosis in colorectal cancer

Shengjie Feng[1], Zejun Rao[1], Jiakun Zhang[1], Xiaowei She[1], Yaqi Chen[1], Kairui Wan[1], Haijie Li[1], Chongchong Zhao[2], Yongdong Feng, Guihua Wang, Junbo Hu, Xuelai Luo[1#]

1, GI Cancer Research Institute, Tongji Hospital, Huazhong University of Science and Technology, Wuhan, 430030, P. R. China.

2, The HIT Center for Life Sciences, Harbin Institute of Technology, Harbin, 150001, China.

# To whom all correspondence should be sent:

Xuelai Luo, GI Cancer Research Institute, Tongji Hospital, Huazhong University of Science and Technology, Wuhan, 430030, China; E-mail: Luoxl@tjh.tjmu.edu.cn.

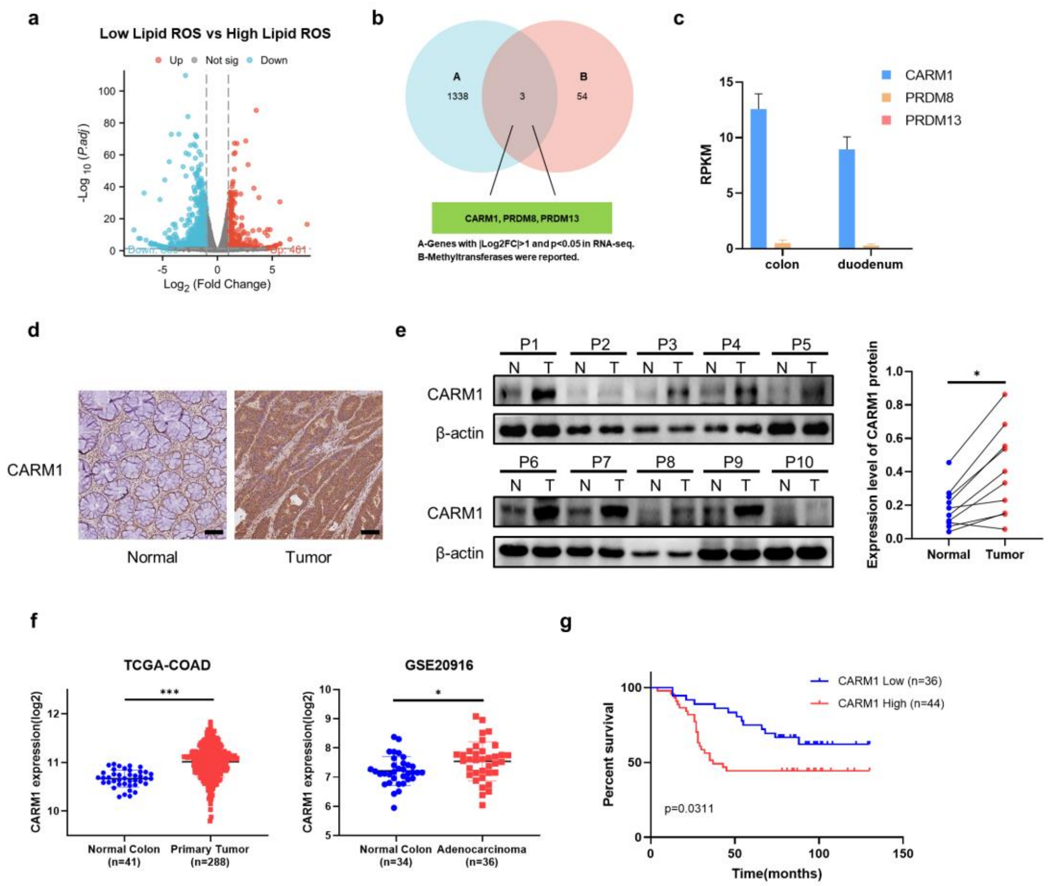

Supplement Figure 1. CARM1 is overexpressed in colorectal cancer. a Volcano plot depicting the gene expression levels in CRC tissues. Relative gene expression levels (fold change, FC) in the low lipid ROS group versus high lipid ROS group are plotted on the x axes as mean log2 ratios (log2(FC)). Log10 transformed p values are plotted on the y axes (log10(p value)). Vertical and horizontal lines indicate fold change and significance threshold criteria of |log2(FC)| >1 and $p < 0.05$. **b** Screening strategy for predicting the possible moderator of ferroptosis. **c** The expression levels of CARM1, PRDM8 and PRDM13 in NCBI database. **d** Representative results of immunohistochemical staining for CARM1 from paired clinical CRC patients. Scale bars, 20 μm. **e** western blot analysis verifying the CARM1 expression in paired clinical CRC tissues. **f** CARM1 expression level in TCGA-COAD dataset and public colorectal cancer expression profile (GSE20916). **g** Kaplan-Meier overall survival

analysis based on IHC of CARM1 in tissue microarry of 80 clinical CRC patients. (n=80, log-rank test). p = 0.0311. The data shown represent mean ± SD. In **e**, comparisons were made using the paired Student's t test. In **f**, comparisons were made by using the two-tailed, unpaired Student's t test; *$p < 0.05$, ***$p < 0.001$.

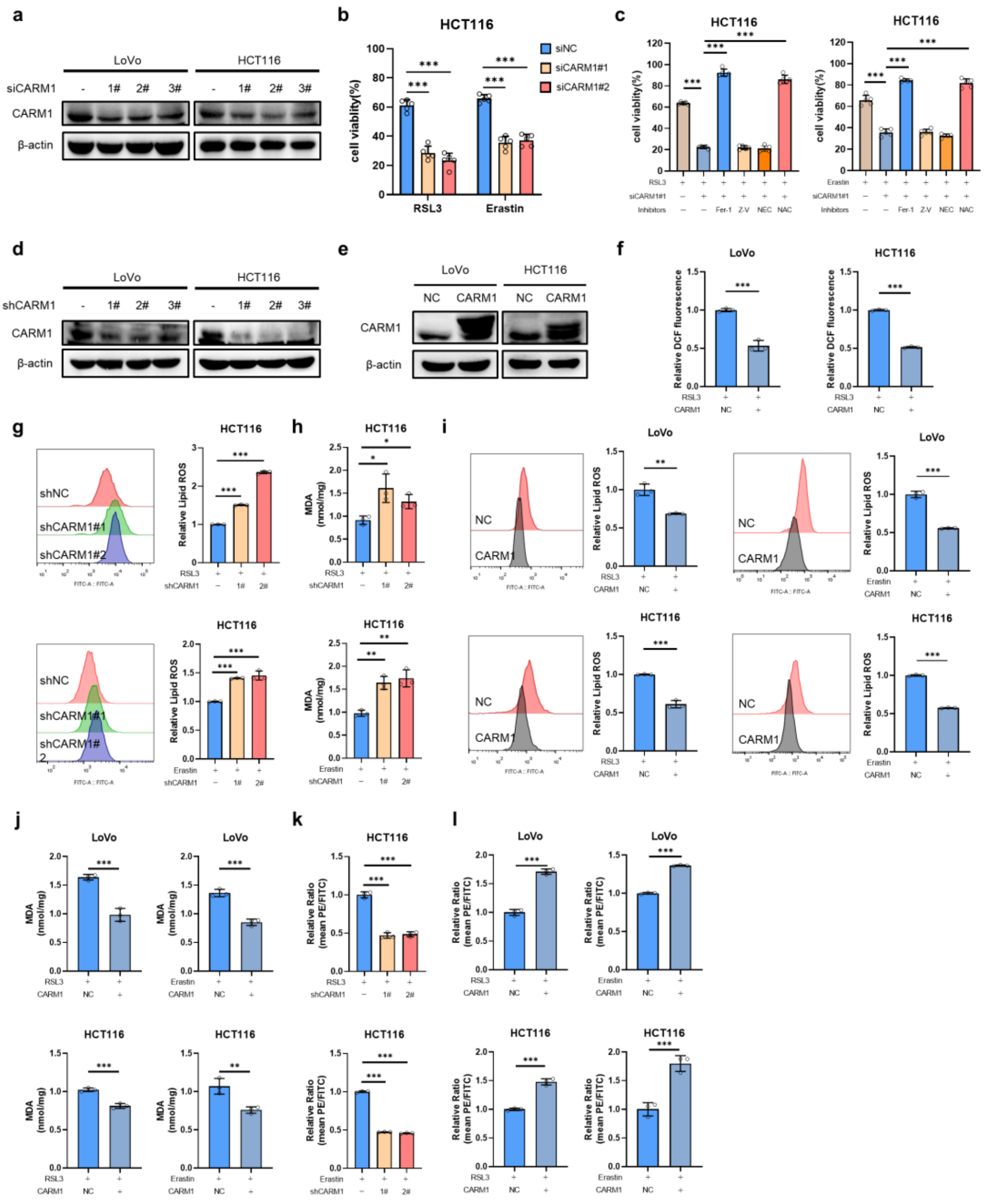

Supplement Figure 2. CARM1 moderate ferroptosis in CRC cells. a western blot analysis verifying the knockdown of endogenous CARM1 via siRNA in LoVo and

HCT116 cells. **b** Cell viability was measured in siNC and siCARM1 HCT116 cells treated with 2.5 μM RSL3 or 5 μM erastin for 12 h. (n=5 independent experiments). **c** Cell viability was measured in siNC and siCARM1 LoVo cells treated with cell death inhibitors and 2. 5 μM RSL3 or 5 μM erastin for 12 h. Ferr-1, 1 μM ferrostatin-1; NAC, 5 mM; Nec, 2 μM necrostatin-1s; Z-V, 20 μM Z-VAD-FMK. (n=5 independent experiments). **d** western blot analysis verifying the knockdown of endogenous CARM1 via shRNA in LoVo and HCT116 cells. **e** western blot analysis verifying the overexpression of CARM1 in LoVo and HCT116 cells. **f** Relative cellular ROS were detected by DCFDA in the vector and CARM1-overexpressing LoVo and HCT116 cells treated with 2. 5 μM RSL3 for 12 h. (n=3 independent experiments). **g-j** Relative lipid ROS and MDA levels were assayed in indicated LoVo cells treated with 2. 5 μM RSL3 or 5 μM erastin for 12 h. (n=3 independent experiments). **k, l** Mitochondrial membrane potential was detected for the indicated cells by fluorescence staining of mitochondria with the JC- 1 dye. (n=3 independent experiments). The data shown represent mean ± SD. In **i- l**, comparisons were made by using the two-tailed, unpaired Student's t test; all other comparisons were made by one-way ANOVA with Tukey's test; $^{**}p < 0.01$, $^{***}p < 0.001$.

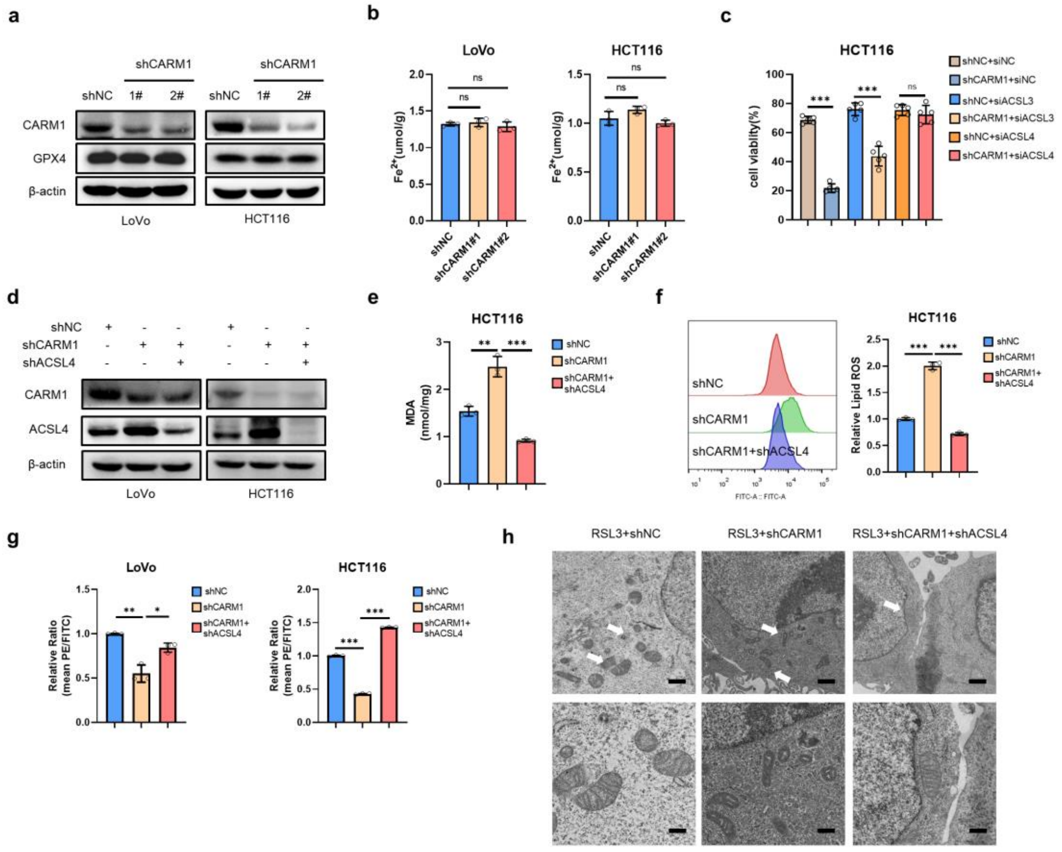

Supplement Figure 3. CARM1 did not affect the protein level of GPX4 or cellular divalent iron levels. **a** western blot analysis of CARM1 KD LoVo and HCT116 cells. Protein levels of CARM1 and GPX4 were assayed. **b** Cellular divalent iron levels were assayed in the indicated LoVo and HCT116 cells. (n=3 independent experiments). **c** Cell viability was assayed in indicated HCT116 cells treated with 2. 5 μM RSL3 for 12 h. (n=5 independent experiments). **d** western blot analysis verifying the knockdown of endogenous CARM1 and (or) ACSL4 via shRNA in LoVo and HCT116 cells. **e, f** MDA levels and relative lipid ROS were assayed in indicated HCT116 cells treated with 2. 5 μM RSL3 for 12 h. (n=3 independent experiments). **g** Indicated stable LoVo and HCT116 cells were used to evaluated mitochondrial membrane potential by fluorescence staining of mitochondria with the JC- 1 dye. (n=3 independent experiments). **h** TEM images of the indicated stable LoVo cells subjected to RSL3 (2.5 μM) for 12 h. white arrows indicate mitochondria.Scale bars: top, 2 μm; bottom, 500 nm. The data shown represent mean ± SD. Comparisons were made by

using one-way ANOVA with Tukey's test; *$p < 0.05$, **$p < 0.01$, ***$p < 0.001$; n.s., no statistic difference.

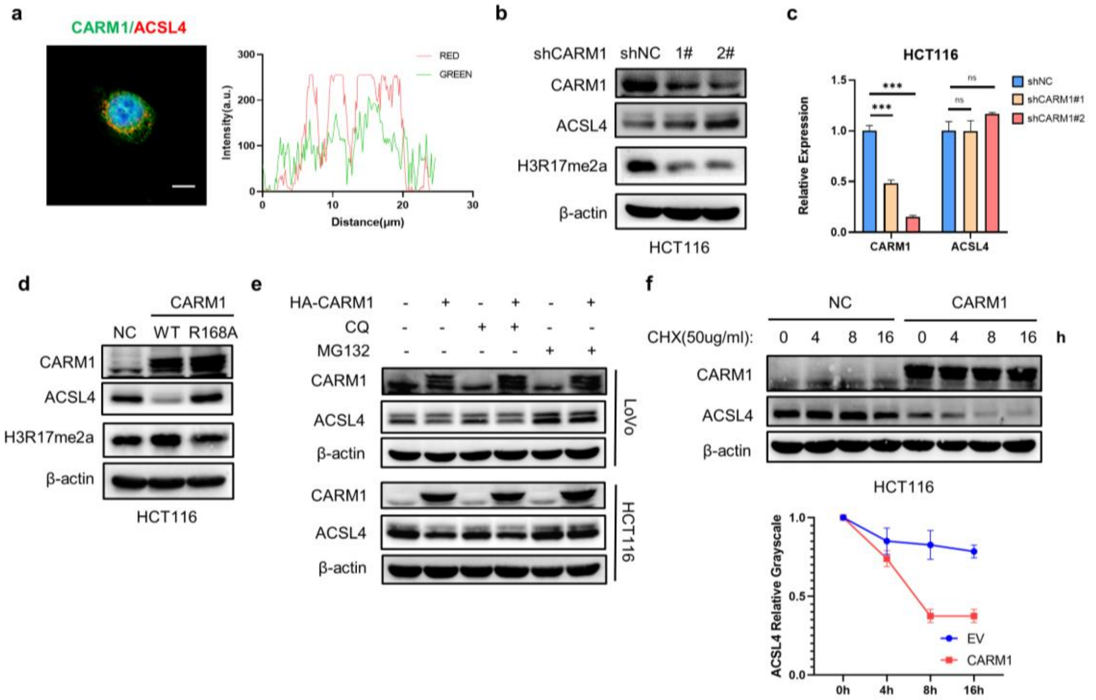

Supplement Figure 4. CARM1 induces ACSL4 degradation in a ubiquitin-proteasome-dependent manner. a Plot profile qualitative analysis was used to describe the co-localization status of CARM1 and ACSL4. **b** western blot analysis of indicated HCT116 cells. Protein levels of CARM1, ACSL4, and H3R17me2a were assayed. **c** qPCR analysis of CARM1 and ACSL4 mRNA levels in indicated HCT116 cells. (n=3 independent experiments). **d** western blot analysis of indicated HCT116 cells. Protein levels of CARM1, ACSL4, and H3R17me2a were assayed. **e** western blot analysis of vector and CARM1 overexpressed LoVo and HCT116 cells treated with treated with DMSO,10 μM MG132 or 25 μM CQ for 8 h. Protein levels of CARM1 and ACSL4 were assayed. **f** western blot analysis of vector and CARM1-overexpressing HCT116 cells treated with 50 μg/mL cycloheximide for the indicated times. Quantitative analysis was conducted on ACSL4 level at indicated time points. The data shown represent mean ± SD. Comparisons were made by using one-way

ANOVA with Tukey's test; ***$p < 0.001$; n.s., no statistic difference.

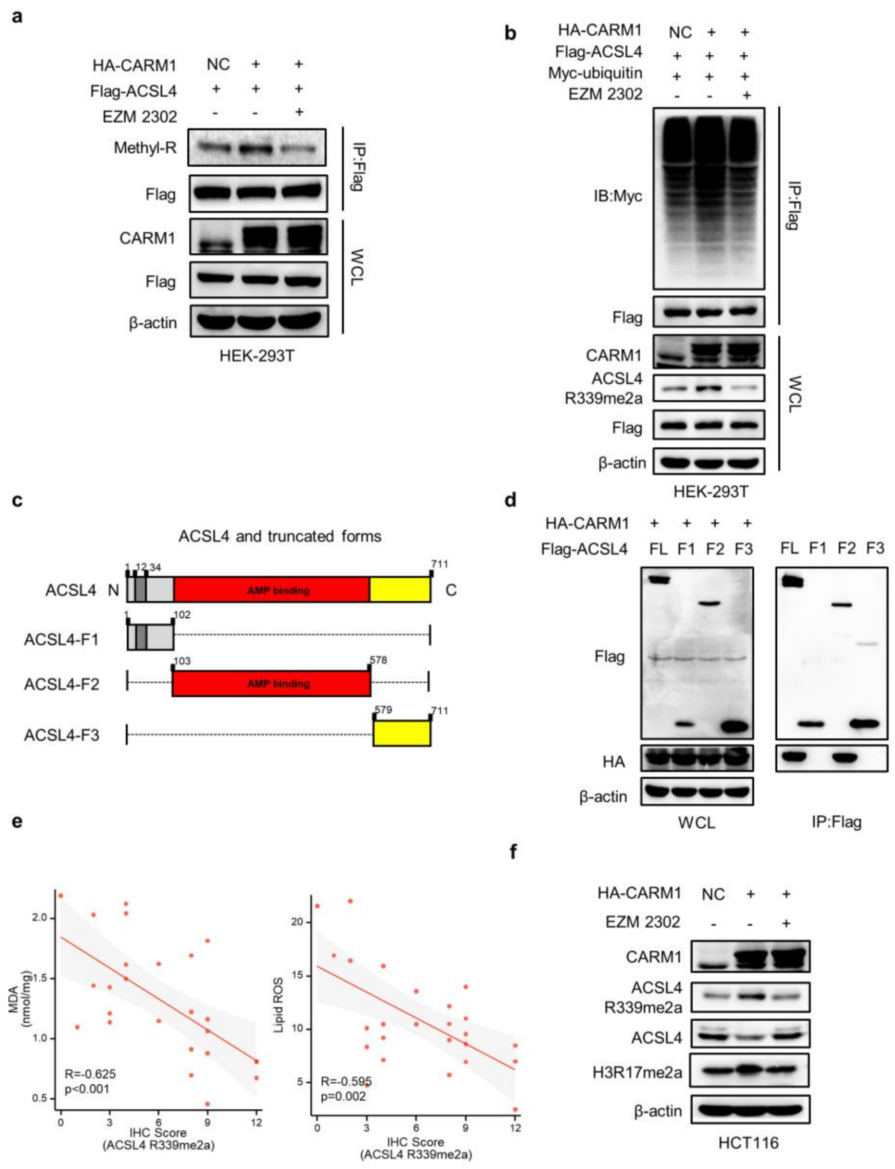

Supplement Figure 5. EZM2302 inhibit CARM1-induced ACSL4 methylation and ubiquitylation. a Co-IP was performed to detect the methylation levels of ACSL4 with CARM1 upregulation treated with EZM2302. **b** HEK293T cells transfected with the indicated plasmids and treated with or without 10nM EZM2302 for 24 h. IP with anti-Flag antibody and western blot with anti-Myc antibody were performed to detect the ubiquitination level of ACSL4. **c** The full-length ACSL4 was divided into three parts and three fragment plasmids were constructed. **d** IP assay was

performed to examine the endogenous interaction between CARM1 and ACSL4 fragments using antibodies against Flag in HEK293T cells. **e** Correlation between ACSL4 R339Ame2a expression and lipid ROS or MDA levels in CRC tissues was determined by Spearman correlation coefficient test. All p and R values were calculated with Spearman's r test. **f** western blot analysis of vector and CARM1-overexpressing HCT116 cells treated with DMSO or 10 nM EZM2302 for 24 h. Protein levels of CARM1, ACSL4 and ACSL4 R339me2a were assayed.

.

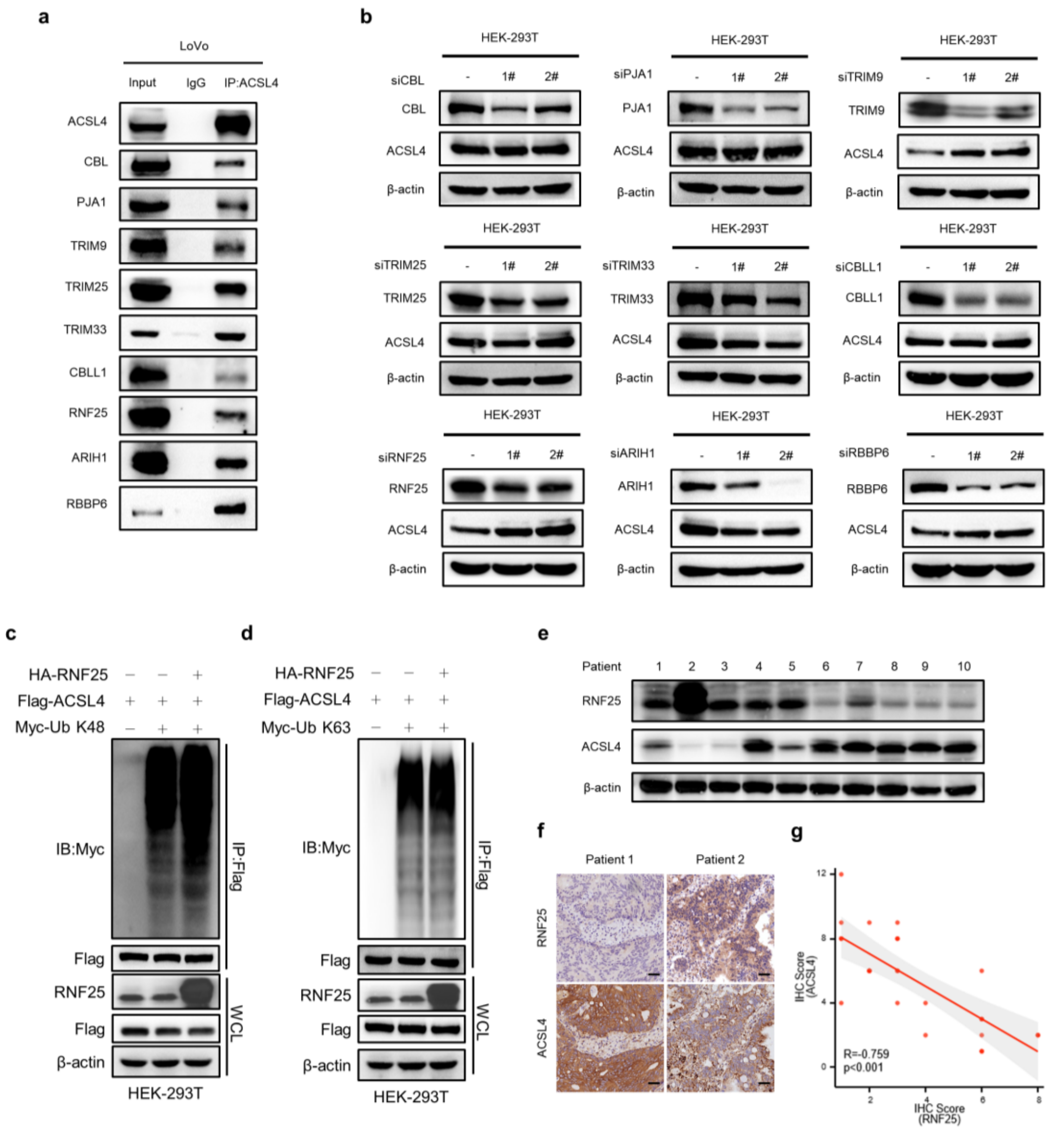

Supplement Figure 6. TRIM9,RNF25 and RBBP6 were potential E3 ubiquitin

ligase of ACSL4. a IP analyses were performed to examine the interaction between indicated E3 ubiquitin ligase and endogenous ACSL4 using antibodies anti-ACSL4 in LoVo cells. **b** western blot analysis of HEK293T cells transfected with indicated siRNAs of E3 ubiquitin ligase.Protein levels of ACSL4 were respective assayed. **c, d** HEK293T cells transfected with the indicated plasmids and treated with 10 μM MG132 for 8 h. IP with anti-Flag antibody and western blot with anti-Myc antibody were performed to detect the ubiquitination level of ACSL4. **e** western blot analysis was performed in paired clinical CRC tissues. Protein levels of RNF25 and ACSL4 were assayed. **f** Representative results of IHC staining for RNF25 and ACSL4 from 25 clinical CRC patients. Scale bars, 20 μm. **g** Correlation between RNF25 and ACSL4 expression in CRC tissues (n=25) was determined by Spearman correlation coefficient test. All p and R values were calculated with Spearman's r test.

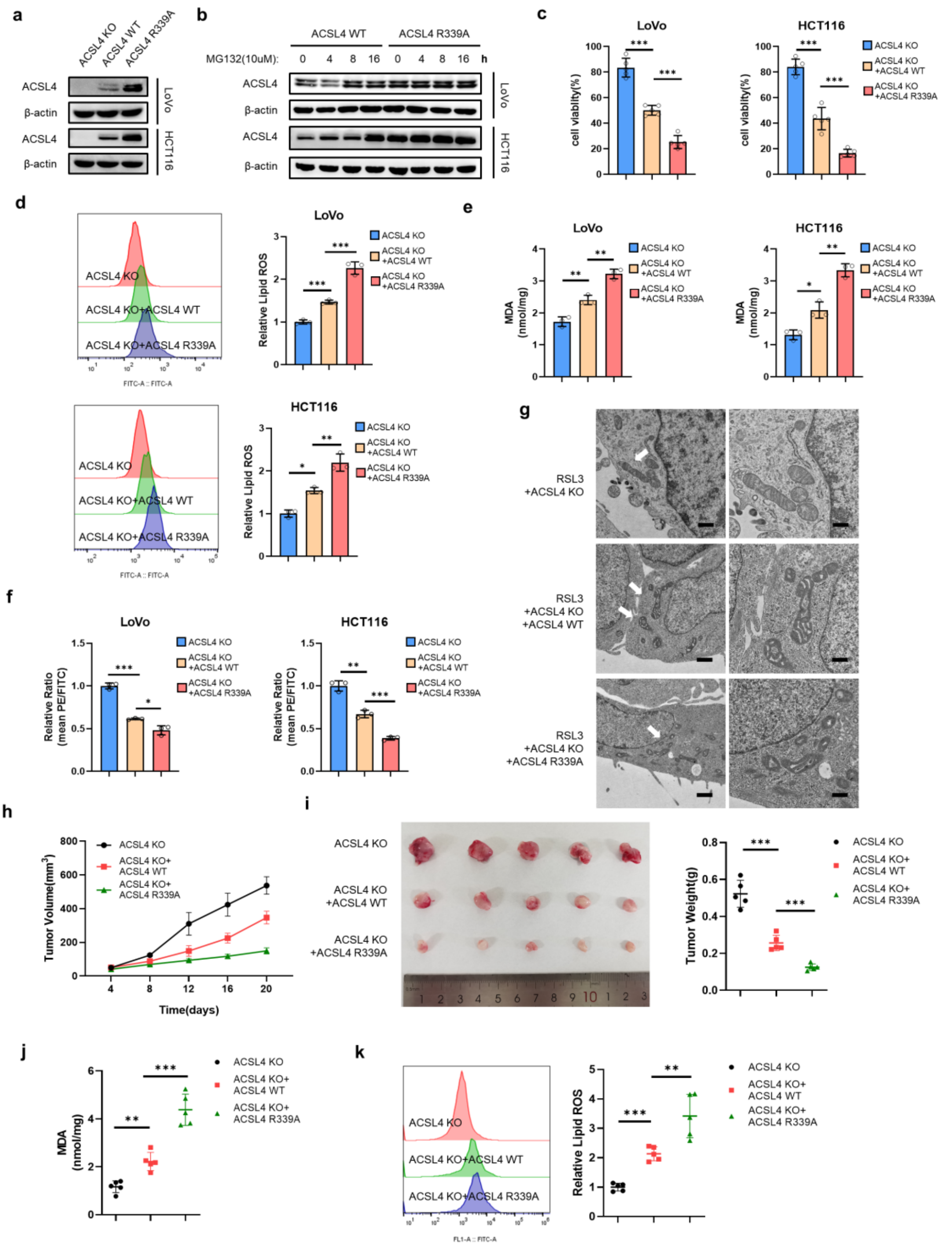

Supplement Figure 7. Inhibition of methylation at R339 of ACSL4 aggravates ACSL4-induced ferroptosis *in vitro* and *in vivo*. a western blot analysis of ACSL4 WT and ACSL4 R339A stable LoVo and HCT116 cells. Protein levels of ACSL4 were respective assayed. **b** western blot analysis of ACSL4 WT and ACSL4 R339A stable LoVo cells treated with 10 μM MG132 for the indicated times. **c** Cell viability was assayed in indicated stable LoVo and HCT116 cells treated with 2.5μM RSL3 for 12

h. (n=5 independent experiments). **d,e** Relative lipid ROS and MDA levels were assayed in indicated stable LoVo and HCT116 cells treated with 2. 5 μM RSL3 for 12 h. (n=3 independent experiments). **f** Indicated LoVo and HCT116 cells treated with 2. 5 μM RSL3 for 12 h and were used to evaluated mitochondrial membrane potential by fluorescence staining of mitochondria with the JC- 1 dye. (n=3 independent experiments). **g** TEM images of the indicated stable LoVo cells subjected to RSL3 (2.5 μM) for 12 h. white arrows indicate mitochondria.Scale bars: left, 2 μm; right, 500 nm. **h** Indicated stable LoVo cells were subcutaneously injected into the mice and RSL3 was injected intratumorally (100 mg/kg, twice per week). Tumor volumes (n=5) were calculated every four days and the growth curve was drawn. **i** The images of tumors size in different groups was showed and the tumor weight (n=5) of the subcutaneous xenografts were measured. **j,k** MDA levels and relative lipid ROS in tumor cells isolated from (**h**) were assayed. (n=5 independent experiments). The data shown represent mean ± SD. Comparisons were made by using the one-way ANOVA with Tukey's test; *$p < 0.05$, **$p < 0.01$, ***$p < 0.001$.

Table S1. The sequence of siRNAs,shRNAs and sgRNAs

**siRNAs**	**Sequence**
siCARM#1	CCAGTAACCTCCTGGATCT
siCARM#2	GTACACGGTGAACTTCTTA
siCARM#3	CAACAACCTGATTCCTTTA
siCBL#1	TACGTATGAAGCAATGTATAATA
siCBL#2	TGGAGAATTTGATGAAGAAAACT
siPJA1#1	CAGCAATTACCTTGAAGAAGTTC
siPJA1#2	TGGCAAGTTTAAAGATGATAAGC
siTRIM9#1	TTCACTTCAACAGCACATACAAC
siTRIM9#2	GAGGTGATTAAGGAAAATGATCC
siTRIM25#1	GACCTTGAAGGAGGAGATTGAAC
siTRIM25#2	CAGCAAGTTTGACACCATTTATC
siTRIM33#1	ACCTCTATTGCCATGAATTAAGT

siTRIM33#2	AGCTTTTCAGGAGGTGTAAAACA
siCBLL1#1	GACAAGATATAGACCGTATTACC
siCBLL1#2	AGCATGTTTTTGCTATGACTGT
siARIH1#1	CAGTCCATTATCTTTGAGAATAA
siARIH1#2	GTCCAATATCCTGATGCTAAACC
siRNF25#1	CAGCACATATGTGAGAAGATTCC
siRNF25#2	AGGAAATTCTCACAGATAACAAC
siRBBP6#1	TGGGCATAAGCAGGAATCAGAGC
siRBBP6#2	AACTACAAAAGCAATTGATGACT
**shRNAs**	**Sequence**
shCARM#1	CTATGGGAACTGGGACACTTT
shCARM#2	CGATTTCTGTTCCTTCTACAA
shCARM#3	GCAGAACATGATGCAGGACTA
shACSL4#1	CCAGTGTTGAACTTCTGGAAA
shACSL4#2	GCAGGAGAGTATGTATCTCTT
shACSL4#3	GCAGTAGTTCATGGGCTAAAT
**sgRNAs**	**Sequence**
sgACSL4#1	CACC-GGAACAGCAGCCATAAGTGTNGG
sgACSL4#2	CACC-GAGATATCTTGCTTTACCTANGG
sgACSL4#3	CACC-GAATTATCTTACCCCAGTCCNGG

Table S2. The prime used to construct the plasmids

Plasmid	Forward Prime	Reverse Prime
ACSL4-F1	GGAAATCCTACTCGAGGACTACAAAGACCA	AGTCCTCGAGTAGGATTTCCCTGGTCCCAA
ACSL4-F2	1-CGCCACCATGAGTGAAGAAAATGAAATGCA<br>2-GAAGTTACAACTCGAGGACTACAAAGACCA	1-TTTCTTCACTCATGGTGGCGGATCCGAGCT<br>2-AGTCCTCGAGTTGTAACTTCACTAGATCTT
ACSL4-	CGCCACCATGGCAGGAGAGTAT	ACTCTCCTGCCATGGTGGCGG

F3	GTATCTCT	ATCCGAGCT
ACSL4R 305A	CCAGTGTGAAGCAATACCTGG ACTGGGACC	GTCCAGGTATTGCTTCACACT GGCCTGTCA
ACSL4R 339A	CTATGGCTGCGCGATTGGATATT CTTCTCC	AATATCCAATCGCGCAGCCAT AGGTAAAGC
ACSL4R 549A	AAATGGACAAGCGTGGTTTTGC ACTGGTGA	TGCAAAACCACGCTTGTCCAT TTTCATCCA

Table S3. The sequences of the primers for the quantitative RT-PCR

Gene	Forward Prime	Reverse Prime
CARM1	CAGCAGAACATGATGCAGGAC	CGCGTAGATTTTCCGTGCTC
ACSL4	CATCCCTGGAGCAGATACTCT	TCACTTAGGATTTCCCTGGTCC
β-actin	CATGTACGTTGCTATCCAGGC	CTCCTTAATGTCACGCACGAT

Table S4. The information of patients.

ID	Gender	Age	TNM stage
60012862267	Male	58	T3N0M0
60013070520	Male	58	T1N0M0
60012900076	Female	53	T3N2M0
60012869490	Male	56	T3N0M0
60010606316	Female	50	T3N2M0
60012916522	Male	58	T2N0M0
60013060173	Male	59	T3N0M0
60013059777	Female	67	T3N0M0
60012981057	Female	52	T4N1M0
60012822872	Female	54	T3N0M0
1002285701	Male	68	T3N0M0

60012948540	Male	59	T2N1M0
60013041135	Male	71	T4N2M0
60012879119	Female	65	T3N0M0
60012870553	Female	59	T3N1M1
60013038698	Female	66	T3N0M0
60012954028	Male	84	T3N0M0
60012833413	Female	55	T1N0M0
60012997846	Male	63	T3N0M0
60012985242	Female	87	T3N1M0
60013053698	Female	57	T4N0M0
60013059102	Female	72	T4N2M0
60012858202	Female	68	T3N0M0
60012903834	Male	59	T3N0M0
60013074302	Female	76	T4N0M0
